# Integration of Multiple Heterointerfaces in a Hierarchical 0D@2D@1D Structure for Lightweight, Flexible, and Hydrophobic Multifunctional Electromagnetic Protective Fabrics

**DOI:** 10.1007/s40820-023-01179-2

**Published:** 2023-08-25

**Authors:** Shuo Zhang, Xuehua Liu, Chenyu Jia, Zhengshuo Sun, Haowen Jiang, Zirui Jia, Guanglei Wu

**Affiliations:** 1 Institute of Materials for Energy and Environment, State Key Laboratory of Bio-Fibers and Eco-Textiles, College of Materials Science and Engineering, Qingdao University, Qingdao, 266071 People’s Republic of China; 2 College of Chemistry and Chemical Engineering, Qingdao University, Qingdao, 266071 People’s Republic of China

**Keywords:** Electrostatic spinning, MOFs, Bimetallic selenide, Hierarchical structures, Multiple heterointerfaces, Electromagnetic wave absorption

## Abstract

The development of wearable multifunctional electromagnetic protective fabrics with multifunctional, low cost, and high efficiency remains a challenge. Here, inspired by the unique flower branch shape of “Thunberg’s meadowsweet” in nature, a nanofibrous composite membrane with hierarchical structure was constructed. Integrating sophisticated 0D@2D@1D hierarchical structures with multiple heterointerfaces can fully unleash the multifunctional application potential of composite membrane. The targeted induction method was used to precisely regulate the formation site and morphology of the metal–organic framework precursor, and intelligently integrate multiple heterostructures to enhance dielectric polarization, which improves the impedance matching and loss mechanisms of the electromagnetic wave absorbing materials. Due to the synergistic enhancement of electrospinning-derived carbon nanofiber “stems”, MOF-derived carbon nanosheet “petals” and transition metal selenide nano-particle “stamens”, the Co_x_Se_y_/NiSe@CNSs@CNFs (CNCC) composite membrane obtains a minimum reflection loss value (RL_min_) of -68.40 dB at 2.6 mm and a maximum effective absorption bandwidth (EAB) of 8.88 GHz at a thin thickness of 2.0 mm with a filling amount of only 5 wt%. In addition, the multi-component and hierarchical heterostructure endow the fibrous membrane with excellent flexibility, water resistance, thermal management, and other multifunctional properties. This work provides unique perspectives for the precise design and rational application of multifunctional fabrics.

**Supplementary Information:**

The online version contains supplementary material available at 10.1007/s40820-023-01179-2.

## Highlights

- Electrospinning combined with structural engineering to construct 0D@2D@1D hierarchical heterogeneous interfaces.
- A “mechanical response” model was constructed to explain the stress transfer mechanism of fibrous membranes with 2D@1D structures.
- The “One for All” fibrous membrane combines excellent flexibility, mechanical properties, waterproof, and electromagnetic protection.

**Supplementary Information:**

The online version contains supplementary material available at 10.1007/s40820-023-01179-2.

## Graphical abstract

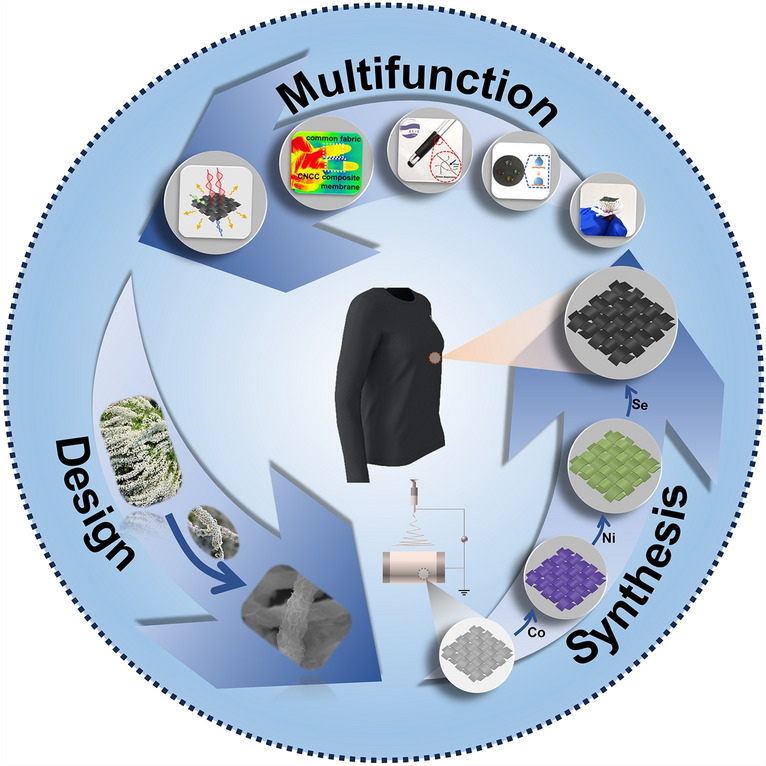

## Introduction

For decades, microelectronic products with mechanical adaptability, portability, lightweight, and waterproof ability have gradually evolved into the best option for emerging integrated circuit equipment. In particular, the fabric products with the characteristics of flexibility, breathability, ease of processing, wear resistance, and low cost have become research hotspots in the area of wearable electronic devices and electronic skin [1–4]. With the rapid development of integrated circuit technology, wearable electronic products are playing an increasingly important role in aerospace, artificial intelligence, Internet of Things, and other fields. However, highly integrated wearable devices also bring substantial electromagnetic pollution, which not only causes serious electromagnetic interference between equipment, but also endangers the health and safety of people and their property [5–7]. During the last several decades, a variety of materials have been investigated as potential electromagnetic protective materials in an effort to reduce this damage, including magnetic metals and oxides [8], polymer materials [9], carbon materials [10], and ceramic materials [11]. Although these materials have achieved satisfactory electromagnetic protective performance to a certain extent, they also have some significant drawbacks such as expensive cost, challenging processing, and poor mechanical adaptability, which are contrary to the development concept of wearable electronic devices [12–14]. Fortunately, these issues may be resolved to the maximum extent possible by optimizing the allocation of components and improving the structural design. Especially, constructing electromagnetic protective fabrics using electrospun-derived carbon nanofibers as the structural substrate and through precise hierarchical heterogeneous structural design can provide multiple interfaces to trigger interfacial polarization, effectively consuming electromagnetic waves with the synergy of various loss processes. The presence of fibers skeletons can also provide necessary flexibility and mechanical properties for composite fabrics. In a nutshell, the design of a highly ordered graphitic carbon matrix with hierarchical structures and the selection of substances with multifunctional application capabilities are essential for the preparation of unique electromagnetic protective fabrics with low density and thin thickness [15–17].

To date, the metal–organic frameworks (MOFs) composed of metal atoms and organic ligands through classical coordination bonds are used extensively in energy storage [18], catalysis [19], gas separation [20], and sensors [21] because of the large specific surface area, adjustable porosity, and customizable microstructure. Due to the highly tunable microstructures and components, MOF derivatives are equally well suited for the development of novel electromagnetic wave absorbing (EMA) materials [22–24]. In general, the organic ligands in MOFs are pyrolyzed to graphitized carbon shells via the high-temperature calcination treatment under inert atmosphere, which contribute to conduction losses, while metal ions can be reduced to metal nanoparticles, leading to additional magnetic losses. However, due to the adverse effects of the one-time high temperature and intense pyrolysis conditions, the microstructure of carbonized derivatives of MOFs with simple spatial structure tends to collapse and decompose inevitably, which impedes free electron transport to some extent. Moreover, the reduced metal nanoparticles also exhibit poor environmental stability due to the high activity in air, which usually leads to absorbers with shorter lifespans and higher maintenance costs. Transition metal chalcogenides have become a research hotspot due to their narrow energy band gap, outstanding electron transfer efficiency, high dielectric constant, and eximious stability [25, 26]. Especially, ternary MOF derivatives have been widely reported in the field of EMA due to the presence of two distinct metal cations in the crystal, which have more active sites and higher conductivity compared with binary MOF derivatives. For example, Wang et al. [27] prepared CoFe-MOF derived Co_7_Fe_3_/C and Co_9_S_8_/FeCoS_2_/C composites by hydrothermal method and subsequent high temperature carbonization and sulfide processes. The Co_9_S_8_/FeCoS_2_/C composite demonstrated excellent EMA performance, and the RL_min_ value of − 53.9 dB was achieved at 20 wt% filling. In addition, it is found that the dielectric properties of the material could be significantly adjusted by altering the type of anion coordination. The advantage of low electronegativity allows selenium to react with metals to form fast electron transfer channels, resulting in higher electrical conductivity and dielectric loss in metal selenides [28]. Furthermore, the variety of nanoparticles generated by bimetallic materials expands, leading to an abundance of non-uniform interfaces, which greatly promote polarization losses [29]. It is worth noting that the introduction of nickel into Co-MOF makes it possible to form bimetallic co-doped MOFs and introduce multiple heterogeneous interfaces in the composite system because of its physicochemical properties and the close proximity of its atomic radius to that of cobalt. The excellent properties of ternary selenides generated from MOFs facilitate the attenuation and consumption of electromagnetic waves.

Another factor that limits the large-scale practical application of MOF derivatives as electromagnetic wave absorbers is the poor processability caused by their severe self-agglomeration. Engineering-wise, the solution to the self-agglomeration issue lies in precisely regulating the growth sites and structure of MOF precursors, as well as in rationally adjusting the subsequent processing technology. The self-agglomeration of MOF materials during high-temperature pyrolysis can be significantly reduced by anchoring the 2D flake MOF precursors on nickel foam, melamine foam, or fiber matrix, thereby ensuring the essential EMA performance of the composites [30, 31]. For example, Liu’s group [32] used a multi-step strategy to efficiently synthesize MOF-derived C@Co/NC@PPy composite fibers, which exhibited excellent EMA performance at a high fill loading of 35 wt%. The composites are made by in-situ growth of MOF precursors using conductive structural substrates, which can both inhibit the self-agglomeration behavior of MOF derivatives and provide additional conduction loss. During the pyrolysis process, organic fibers can be transformed into carbon fibers to maintain excellent structural stability, and form abundant heterogeneous interfaces with MOF derivatives to induce interfacial polarization. Furthermore, the highly porous structure, large specific surface area, and designable micromorphology of the MOF-derived materials themselves can confer a variety of capabilities beyond the EMA properties of the composites [33–35].

In this work, we propose a rational hierarchical structure design for the most typical zeolite imidazolate framework material (ZIFs) of MOF materials and obtain autocatalytic pyrolysis carbon fibers loaded with MOF derived selenides through component adjustment and structural optimization, enabling the realization of electromagnetic protective fabrics with multifunctional advantages. Precise morphological regulation enables the construction of multifunctional “one for all” 0D@2D@1D structure, a unique hierarchical nanostructure combining zero-dimensional (0D) selenide nanoparticles (SNPs), one-dimensional (1D) carbon nanofiber frameworks (CNFs) and two-dimensional (2D) carbon nanosheets arrays (CNSs), forming a unique structure similar to the morphology of flower branch shape of “Thunberg’s meadowsweet” in nature. Particularly due to the continuous and interconnected structure between 1D nanofibrous and 2D nanoarrays, it is possible to considerably optimize the electron transport channels, provide sufficient spatial gaps and sites for multiple reflections and scattering of electromagnetic waves inside the absorber and contribute to multiple heterogeneous interfaces for triggering enhanced polarization losses. Based on the above benefits, the fibrous composite outperforms conventional MOF-derived composites and carbon fiber-based absorbers in terms of EMA performance. Additionally, by properly combining fiber carriers and MOF derivatives, fibrous composites can achieve lots of other functions, including strong hydrophobicity, high air permeability, extraordinary flexibility, excellent mechanical adaptability, and certain thermal management capabilities. This work provides a valuable reference for the precise design of multifunctional fabrics with MOF-derived hierarchical structures.

## Experimental Section

### Materials

Cobalt (II) nitrate hexahydrate (Co(NO_3_)_2_·6H_2_O, AR), nickel (II) nitrate hexahydrate (Ni(NO_3_)_2_·6H_2_O, AR), 2-methylimidazole (2-MI, C_4_H_6_N_2_, AR) were purchased from Sinopharm Chemical Reagent Co., Ltd. N,N-Dimethylformamide (C_3_H_7_NO, DMF, AR) was purchased from Shanghai Aladdin Biochemical Technology Co., Ltd. Selenium powder (Se, 99%) and polyacrylonitrile (PAN, $\overline{Mw }$ = 150,000, 99%) were purchased from Shanghai Macklin Biochemical Co., Ltd. All chemicals were of analytical grade and used without further purification.

### Preparation of Co-ZIF/PAN

Co-ZIF/PAN fibrous membrane was prepared by electrostatic spinning and impregnation growth of ZIF nanoarrays. Briefly, 0.2 g of 2-methylimidazole and 1 g of PAN were weighed and dissolved in 10 mL of DMF and stirred at 60 °C for 12 h to obtain a precursor solution for electrospinning. Then, the precursor solution was placed in a 10 mL syringe with a 17G electrospinning needle. During the electrospinning process, the working voltage, syringe propelling speed, drum rotation speed, and the distance between the spinneret and the collector were fixed at 18 kV, 0.9 mL h^−1^, 500 r min^−1^, and 20 cm, respectively (at room temperature of 25 °C, 40% humidity). The obtained precursor nanofibrous membrane was cut into small pieces with a size of 2 × 2 cm^2^ for subsequent growth of Co-ZIF.

The growth of Co-ZIF nanosheet arrays on PAN nanofibers were performed by direct impregnation at conventional conditions. Two solutions were prepared separately by dissolving 0.5 mmol of Co(NO_3_)_2_·6H_2_O and 4 mmol of 2-methylimidazole in 10 mL deionized water. Then, the Co^2+^ solution was quickly added into the 2-methylimidazole solution and stirred slowly for 1 min to form the ZIF-impregnated growth solution. The precursor nanofibrous membrane was subsequently immersed vertically in the growth solution and aged for 4 h. Finally, the purple fibrous membrane was taken out and washed three times with deionized water, and dried in a vacuum oven at 70 °C for 12 h. The obtained purple nanofibrous membrane with Co-ZIF anchored on the surface was named Co-ZIF/PAN.

### Preparation of CoNi-ZIF/PAN

The CoNi-ZIF/PAN was prepared by cation exchange method. Typically, 1 mmol of Ni(NO_3_)_2_·6H_2_O was dissolve in 20 mL of mixed solution of absolute ethanol and deionized water (volume ratio, ethanol:H_2_O = 1:1) and stirred for 1 h at room temperature to obtain a homogeneous solution. The Co-ZIF/PAN nanofibrous membrane prepared above was placed in Ni^2+^ solution, and was ultrasonically treated at 100 W for 1 h. Afterwards, the fibrous membrane and the solution were transferred to a 50 mL Teflon-lined stainless-steel autoclave and reacted at 120 °C for 1 h. After the reaction was completed, the fibrous membrane was taken out and washed three times with deionized water, and then dried in a vacuum oven at 70 °C for 12 h. The obtained nanofibrous membrane with CoNi-ZIF anchored on the surface was named CoNi-ZIF/PAN.

### Preparation of Co_x_Se_y_/NiSe@CNSs@CNFs (CNCC) Fibrous Composite

The CoNi-ZIF/PAN prepared above was pre-oxidized in a muffle furnace at 250 °C for 1.5 h at a heating rate of 2 °C min^−1^. The pre-oxidized fibrous membrane was then placed in a tube furnace and annealed at 600 °C for 1 h at a heating rate of 5 °C min^−1^ under an argon atmosphere to obtain a sheet-like ZIF-derived CoNi-carbon hybrid-modified carbon nanofibers (CoNi-C@CNFs). After that, the carbonized fibrous membrane was subjected to further selenization.

Specifically, the selenium powder was placed in a porcelain boat upstream of the gas stream in a tube furnace, and the initially carbonized fibrous membrane was placed in another porcelain boat downstream of the gas stream in a tube furnace, keeping a gap of 3 cm between them. Subsequently, the fibrous membrane was annealed at 700 °C for 1 h under a mixed H_2_/Ar atmosphere (H_2_ content 5%) with a heating rate of 5 °C/min to obtain Co_x_Se_y_/NiSe@CNSs@CNFs (CNCC). The weight ratio of selenium powder to the initial carbonization-treated fibrous membrane was 1:1. In addition, Co–C@CNFs and CoSe@CNSs@CNFs (CCC) were sequentially obtained by treating Co-ZIF/PAN in the same way.

### Characterization

The crystal structures of all samples were characterized by X-ray diffraction (XRD) (λ = 0.15418 nm). Raman spectra were acquired at 532 nm using a Renishaw InviaPlus micro-Raman spectroscopy system equipped with a 50 mW DPSS laser. The microstructure, morphology, and element distribution of the samples were observed by field emission scanning electron microscope (SEM, JEOLJSM7800F) equipped with energy dispersive spectrometer (EDS) and transmission electron microscope (TEM, JEOLJEM2100). The elemental composition and chemical bond state of the samples were determined by X-ray photoelectron spectroscopy (XPS). The hydrophilic and hydrophobic properties of the sample surface were measured by a contact angle measuring instrument (BOEN-6489, Shanghai Fairborn Industrial Development Co., Ltd., China). Thermogravimetric analysis (TGA) was performed by a simultaneous thermogravimetric analyzer (TGA2, Mettler-Toledo) under an argon atmosphere. Nitrogen adsorption–desorption isotherm measurements were performed at 77 K with an adsorption analyzer (ASAP-2460, Micromeritics). The tensile behavior of the fibrous membranes was measured by an Instron 3300 Universal Testing Systems instrument. The surface resistance of the samples was tested with a multimeter (VICTORVC890D). Specific surface area and pore size distribution of the samples were examined by the Brunauer–Emmett–Teller (BET) method using nitrogen adsorption and desorption isotherms on a physical adsorption analyzer (Quantachrome Autosorb iQ3). The thermal conductivity of various fiber membranes was tested with a portable thermal conductivity meter (TC3000E, 300 K).

### Electromagnetic Parameters

A vector network analyzer (N5234A, Agilent) was used to characterize the electromagnetic parameters at 2 ~ 18 GHz and further analyzed the electromagnetic wave absorption characteristics of the samples. Prior to testing, the prepared samples were uniformly dispersed in the paraffin matrix with a filling amount of 5 wt%. Then, the mixture was pressed into a ring shape (φ_out_: 7.00 mm, φ_in_: 3.04 mm, thickness: 2.0 mm). In general, the electromagnetic absorption characteristics of EMA materials are related to complex permittivity, complex permeability and impedance matching, and the reflection loss can be calculated according to the transmission line theory [36–40]:

1 $$ {\text{RL}}\left( {{\text{dB}}} \right){\text{ = 20lg}}\left| {\frac{{Z_{in} { - }Z_{0} }}{{Z_{in} { + }Z_{0} }}} \right| $$

2 $$ Z_{in} { = }Z_{0} \sqrt {\frac{{\mu_{r} }}{{\varepsilon_{r} }}} {\text{tanh}}\left( {j\frac{2\pi fd}{c}\sqrt {\mu_{r} \varepsilon_{r} } } \right) $$

where Z_in_ and Z_0_ represent the input impedance and free space impedance of the normalized absorber, respectively, while ε_*r*_ and μ_*r*_ represent the complex permittivity and complex permeability, *f* is the frequency of the electromagnetic wave, *c* is the propagation speed of light in free space, and* d* is the thickness of the absorber.

## Results and Discussion

### Composition and Structure

**Fig. 1:**
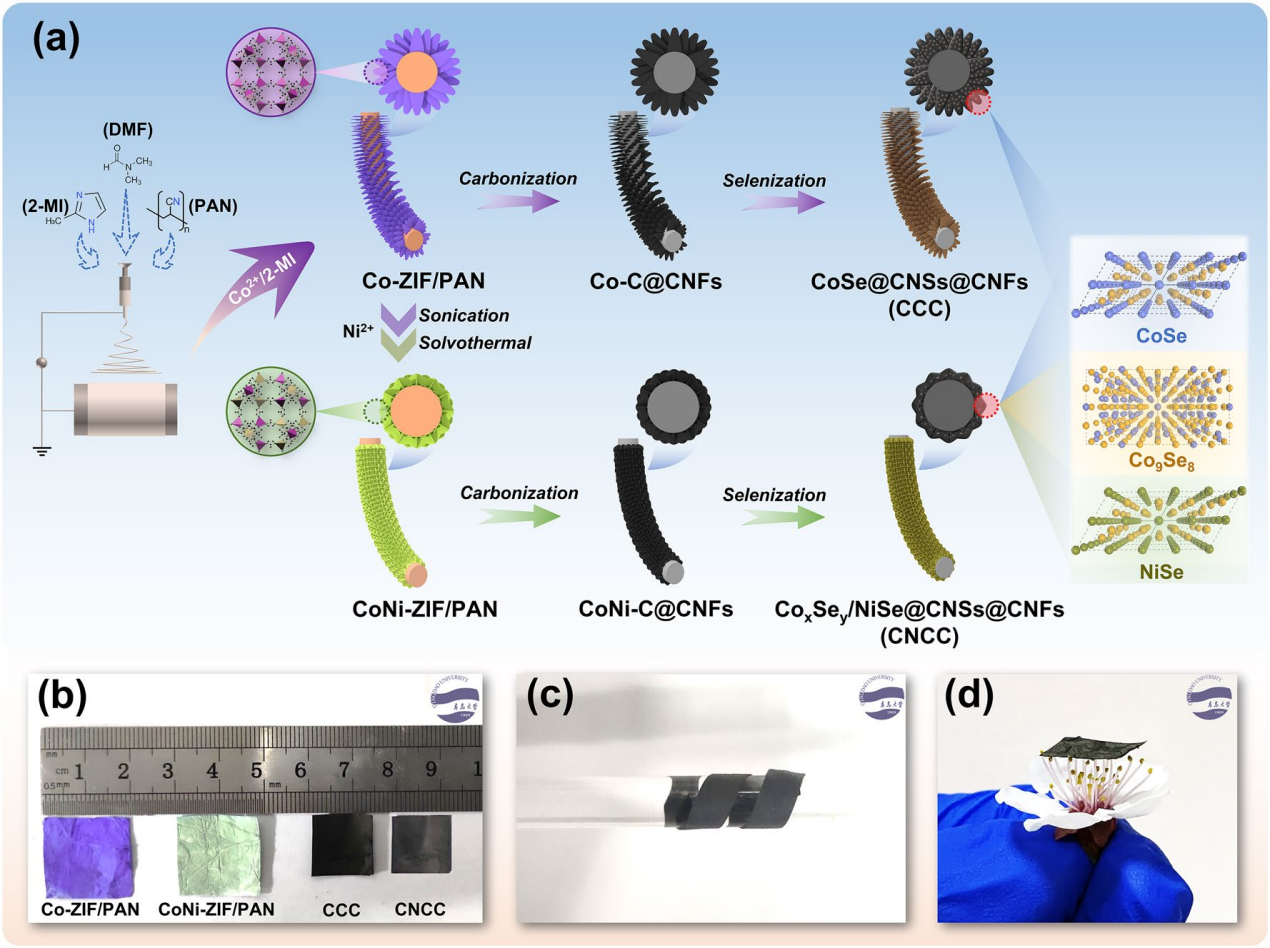
**a** Schematic diagram of the synthesis of CoSe@CNSs@CNFs (CCC) and Co_x_Se_y_/NiSe@CNSs@CNFs (CNCC), **b** digital image of various fibrous membranes **c** flexibility demonstration, and **d** lightweight demonstration of CNCC fibrous composite membrane

Figure 1a schematically shows the synthesis process of CoSe@CNSs@CNFs (CCC) and Co_x_Se_y_/NiSe@CNSs@CNFs (CNCC) fibrous composites. Specifically, an appropriate amount of 2-methylimidazole is first added to the PAN spinning solution, so that it is evenly distributed as a “target” on the electrospun PAN nanofibers. Then Co^2+^ ions in the coordination state are induced to form 2D zeolite imidazole framework (Co-ZIF) nanoarrays on the surface of the nanofibers. Subsequently, using Co-ZIF itself as a template, Ni^2+^ ions are used to replace part of Co^2+^ in the zeolite imidazole framework by a cation exchange method, thereby transforming the monometallic sheet-like Co-ZIF into the bimetallic co-doped petal-like CoNi-ZIF. In detail, the strong coordination between imidazole and transition metal ions, as well as the similar electronegativity and ionic radius of Ni^2+^ and Co^2+^ in the crystal structure of the zeolite imidazole framework, make it possible to incorporate Ni^2+^ into the Co-ZIF framework homogeneously [41, 42]. Finally, after initial carbonization and post selenization, the carbon nanosheet arrays are obtained on the graphitized carbon nanofiber frameworks, in which metal selenide nanoparticles are anchored on the carbon nanosheets. The carbonization treatment maintains the basic structure of the ZIF precursors, which allows them to retain the original morphology after the subsequent selenization treatment. Figure 1b shows the corresponding digital pictures of various fibrous membranes. The dark purple Co-ZIF/PAN fibrous membrane is transformed into the light green CoNi-ZIF/PAN fibrous membrane via the cation exchange process, with little changes in mass and volume. However, the fibrous membrane undergoes about 70% mass reduction and about 30% volume shrinkage after the carbonization and selenization process. A simple test reveals the excellent flexibility of CNCC membrane, and the membrane could be wrapped around a glass rod twice without fracture (Fig. 1c), which means that there are no macroscopic defects in the fibrous membrane that would affect its structural stability. In addition, the CNCC fibrous composite membrane exhibits an ultra-low density of 0.235 g cm^−3^ as a consequence of the autocatalytic pyrolysis of the precursor fibers, which eliminates a large amount of non-carbon components (such as N and O) and reorganizes carbon elements. It turned out that the ultrathin fibrous membrane could be stably placed on a cluster of soft stamens (Fig. 1d). Lightweight and flexible are the necessary basis for the practical application of electromagnetic protective fabrics.

**Fig. 2:**
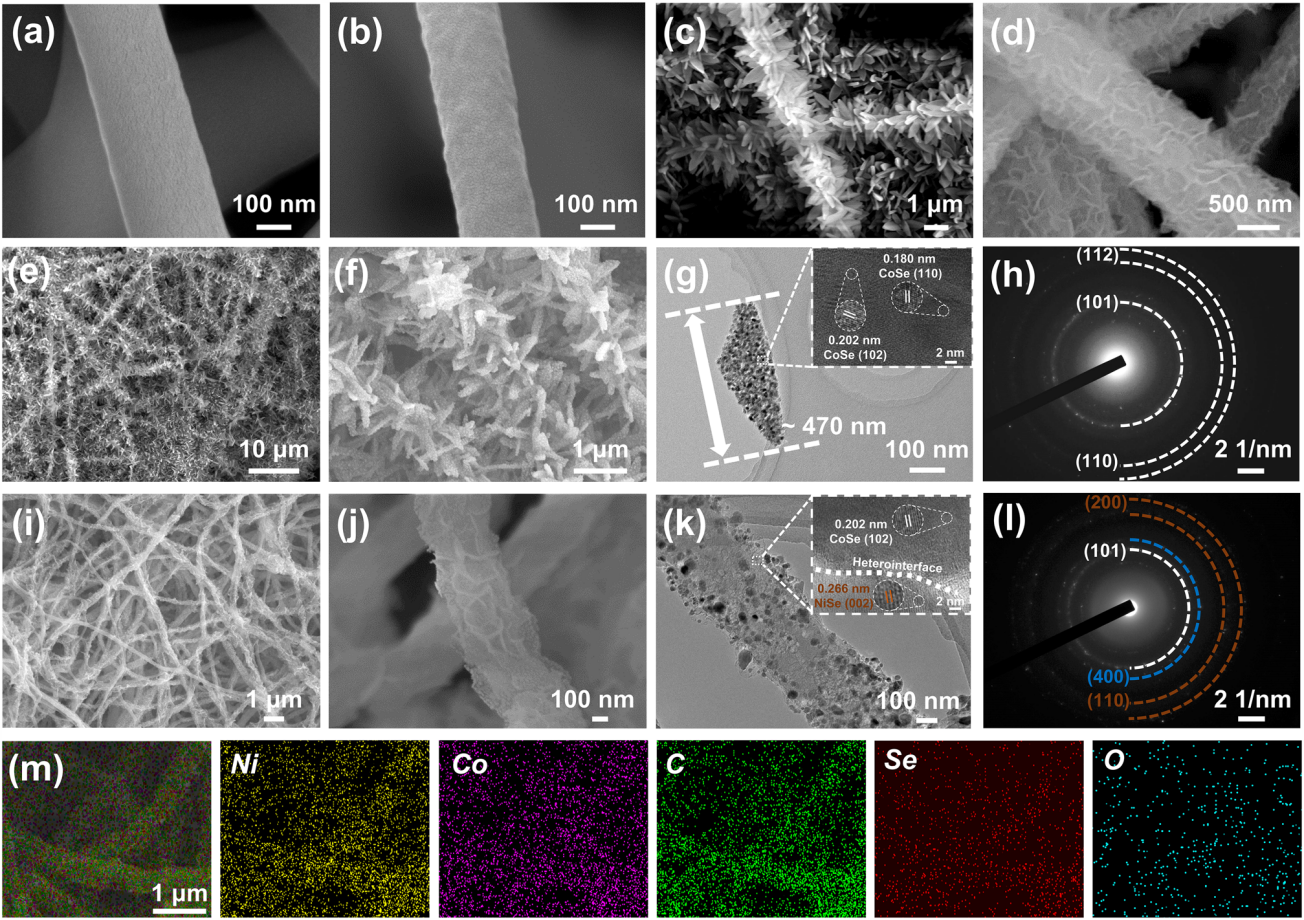
SEM images of **a** PAN fibers, **b** unmodified CNFs, **c** Co–C@CNFs and **d** CoNi-C@CNFs, **e, f** SEM, **g** TEM, **h** SAED image of sample CCC, (i, j) SEM, **k** TEM, **l** SAED image and **m** EDS element mapping of sample CNCC

The microstructure and morphology of the various samples are examined by SEM and TEM (Fig. 2), and the diameter distribution of various fibers is counted using histograms (Fig. S1). The PAN nanofibers exhibit a 1D continuous nanofiber structure with a smooth surface and randomly staggered arrangement (Fig. 2a), with an average diameter of approximately 274 nm (Fig. S1a). The surface roughness of the pure carbon nanofibers obtained without grafted ZIF nanoarrays and only after carbonization increases slightly (Fig. 2b) and the average fiber diameter decreases slightly to approximately 240 nm (Fig. S1b). By targeting and trapping Co^2+^ in the coordinated state, a “bamboo leaf”-like nanosheet structure composed of Co-ZIF uniformly anchored onto the nanofiber frameworks is formed, resulting in Co-ZIF/PAN (Fig. S2a). In the presence of only Co^2+^ in the ZIF-impregnated growth solution without the addition of 2-methylimidazole (Co^2+^:2-MI = 1:0), the prepared Co-ZIF/PAN shows only a small number of amorphous nanosheets scattered on the fiber surface (Fig. S2b). This is since the Co-ZIF nanocrystals are insufficient to create a well-established crystal structure at the low 2-methylimidazole level, making it unable to build nanosheet arrays on the fibers. When the concentration of 2-methylimidazole in the growth solution is excessive (Co^2+^:2-MI = 1:16), Co^2+^ ions preferentially coordinate with 2-methylimidazole in the growth solution to form triangular nanocrystals in a very short period, further accumulating into carambola-like or cruciform-like bulks of varying sizes, thus failing to fully form nanosheet structures on the fiber surface (Fig. S2c). Based on the above results, the ZIF-impregnated growth solution with the optimal condition of Co^2+^ to 2-MI ratio of 1:8 is selected for the growth of Co-ZIF nanoarrays on the surface of nanofibers. During the initial pyrolysis process, the Co-ZIFs grafted on the surface of the PAN fibers are transformed into a Co-doped carbon nanosheets, while the “bamboo leaf”-like structure is well maintained (Fig. 2c). With the assistance of high-power ultrasound and solvothermal treatment, the Co-ZIF nanosheets themselves are used as templates, and Ni ions gradually replaces some of the Co ions in the zeolite imidazole framework through a cation exchange process, forming a Co–Ni co-doped petal-like structure (Fig. S2d). The carbonization process converts the CoNi-ZIFs into Co and Ni co-doped carbon-based nanoflakes, similar to the sample CCC, with little to no change in the fundamental form of the “petals” arranged axially along the fibers (Fig. 2d). A dense coating of selenide nanoparticles developed on the surface of the nano- nano- “leaves” and nano- “petals” grew on the fibers after the post-selenization treatment, and the matrix skeleton is maintained well because of the prior carbonization procedure (Fig. 2e-f and i-j). Evidently, after being subjected to carbonization and selenization, the average fiber diameters of CCC and CNCC somewhat decrease in comparison to Co-ZIF/PAN and CoNi-ZIF/PAN, which directly contributes to the overall shrinking of the fibrous membranes (Fig. S1c-f). The SEM images of Co-ZIF/PAN and CoNi-ZIF/PAN directly subjected to selenization without initial carbonization are shown in Fig. S3a, b, respectively. The high-temperature gas-phase selenization process of metal–organic frameworks is accompanied by the removal of non-carbon elements and the structural reorganization of carbon elements in organic substances, while selenide nanoparticles would generate continuous micro-stress due to self-agglomeration during the forming process. These inevitable micro-stresses could cause a certain degree of disturbance to the formation process of the carbon skeleton, thus destroying the regular structure of the “leaves” and “petals”. The initial carbonization treatment in the early stage maintained the carbon skeleton in an intact and firm state, thereby restricting the self-agglomeration behavior of nanoparticles during the selenization process and simultaneously ensuring the homogeneity of nanoparticle size [43–46]. The existence of carbon fiber fundamental skeleton and carbon nanosheet arrays can form countless regional or global conductive networks, which undoubtedly have a positive impact on the transmission and transfer of electrons. The microstructures of samples CCC and CNCC are further investigated by high resolution transmission electron microscopy (HRTEM). The TEM images confirm the unique hierarchical structure of these two samples, with lots of tiny carbon-coated nanoparticles uniformly decorated on the ultrathin nanosheets on the fiber surface (Fig. 2g, k). Specifically, the size of the nanosheets modified on the carbon fiber surface in sample CCC is approximately 470 nm, while the size of the lamellae in sample CNCC is less than 50 nm. The graphitic layers on the surface of the nanoparticles are attributed to the initial carbonization treatment of the fibrous composites. The metal species in the ZIFs are reduced to metal clusters and migrate to the surface as a result of the reducing gases such NH_3_ and HCN created by the decomposition of polyacrylonitrile at lower temperatures. Eventually, the metal clusters turn into molten nanoparticles as the temperature rises. At the same time, carbon radicals decomposed by organic matter diffuse to the surface of metal nanoparticles to form an outer carbon wall. Selenium elements are introduced into the carbon layer during the post-selenization process via an anion exchange procedure, where they interact with metal nanoparticles to create carbon-coated metal selenide nanoparticles. The HRTEM image in Fig. 2g shows the structure of carbon-encapsulated nanoparticle more clearly, and the lattice fringes with interplanar spacing of 0.202 nm and 0.180 nm correspond to the (102) and (110) crystal planes of CoSe, respectively. The combination of selected area electron diffraction (SAED) image (Fig. 2h) and elemental mapping distribution (Fig. S4) further demonstrates that sample CCC contains only a sort of metal selenide, CoSe, uniformly distributed on the nanosheets and aligned axially along the nanofibers. The HRTEM image of the sample CNCC (Fig. 2k) shows the lattice fringes with interplanar spacing of 0.202 and 0.266 nm corresponding to the (102) plane of CoSe and the (002) plane of NiSe, respectively, and there is an obvious heterogeneous interface between them (marked with a white dotted line). Furthermore, from the SAED image in Fig. 2l, a diffraction ring belonging to the (400) plane of Co_9_Se_8_ is present in the CNCC sample, demonstrating the polycrystalline nature of CNCC. The introduction of nickel element changed the unit cell structure of CoSe, and the spatial arrangement of selenide species also changed, resulting in the appearance of Co_9_Se_8_, which is beneficial to the generation of multiple heterostructures. According to the elemental mapping (Fig. 2m), the CoNi-C@CNFs fibrous membrane has been successfully selenized, producing three different metal selenides, CoSe, Co_9_Se_8_, and NiSe, as well as forming abundant heterointerfaces between various species. The oxygen element spectrum shows that there is a very small amount of oxygen species in the sample, which may be attributed to the slight oxidation of some elements or the existence of adsorbed oxygen on the surface of the sample. The combination of 0D metal selenide particles, 1D fibrous framework, and 2D carbon nanoarrays constitutes a unique hierarchical nanostructure, results in abundant micro-heterointerfaces, which facilitates enhanced interfacial polarization and EMA performance [47, 48].

**Fig. 3:**
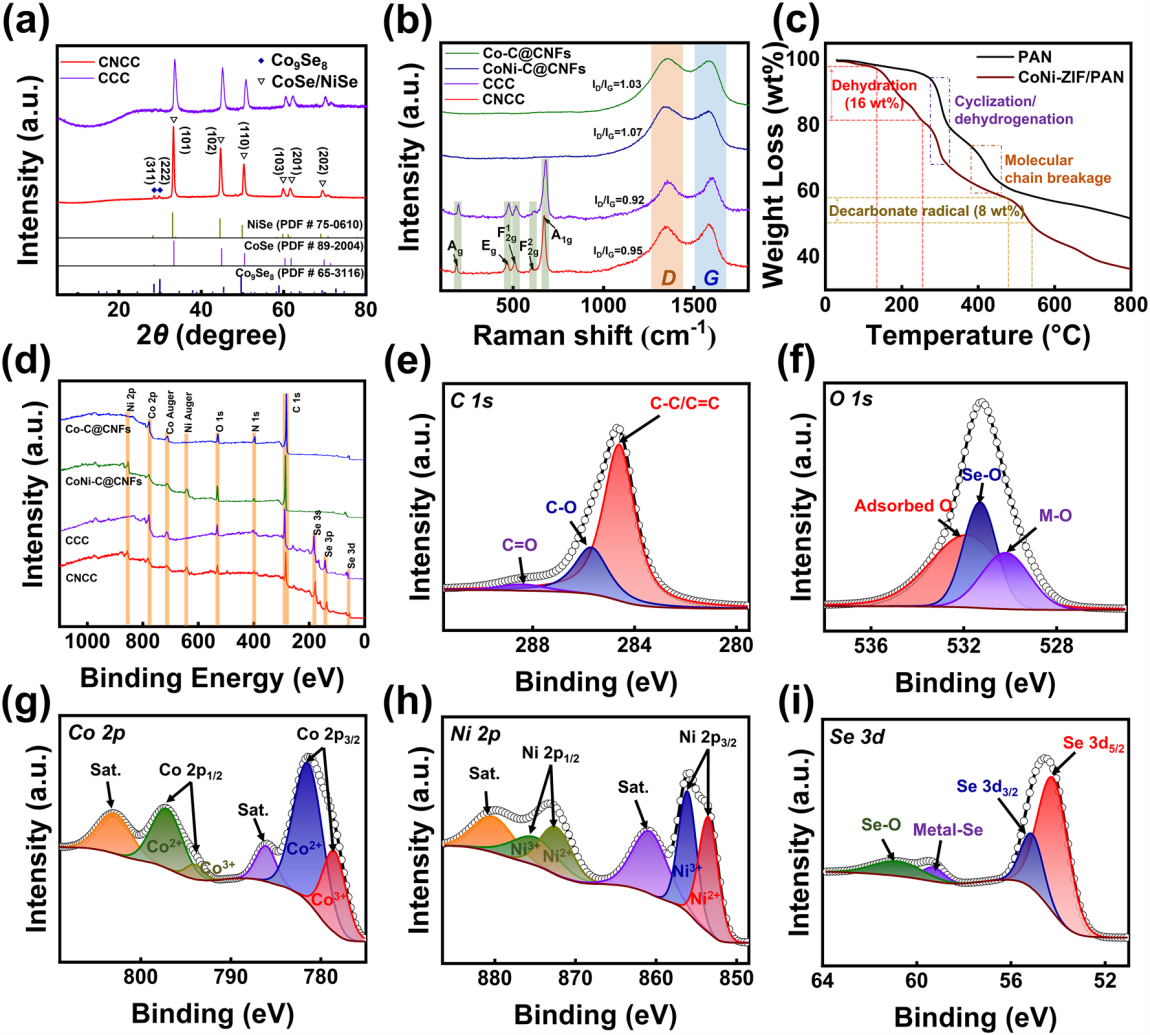
**a** XRD patterns, **b** Raman patterns, **c** TGA curves, **d** total XPS spectra of sample CNCC, and the corresponding high-resolution spectra of **e** C 1s, **f** O 1s, **g** Co 2p, **h** Ni 2p, and **i** Se 3d

The crystal structures and phase composition of various samples are further analyzed by XRD. The phase state of the zeolite imidazole framework structure varies during the cation exchange process, as can be observed from the XRD patterns of Co-ZIF, Ni-ZIF, and bimetallic co-doped ZIF in Fig. S5. The structure of ZIFs is regulated by varying the sonication duration while the solvothermal reaction temperature and time are maintained constant. The sample treated with ultrasound for 1 h contains two relatively complete crystal structures of Co-ZIF and Ni-ZIF, and the amounts of Co and Ni are comparable in zeolite imidazole framework. As a result, CoNi-ZIF-1 h is selected as the sample for subsequent treatment. The positions of the corresponding diffraction peaks on the (101), (102), (110), (103), (201), and (202) crystal planes of hexagonal CoSe (JCPDS No. 89–2004) [49] and hexagonal NiSe (JCPDS No. 75–0610) [50] are highly similar, which corresponds to the XRD patterns of the samples CNCC and CCC (Fig. 3a). This is due to the fact that both CoSe and NiSe belong to a hexagonal crystal system with a space group of *P*6_3_/*mmc* and their lattice constants are quate close, resulting in similar diffraction patterns and crystal structures of the two metal selenides [51]. The only difference is that the most obvious main diffraction peaks in CNCC are slightly shifted to the direction of the lower diffraction angle compared to CCC. The Bragg diffraction formula interprets this as an increase in the lattice constant, which causes a slight rise in the interplanar spacing. In addition, the tiny diffraction peaks around 28.2° and 29.6° of the sample CNCC are attributed to the (311) and (222) planes of Co_9_Se_8_. However, these two diffraction peaks are not shown in XRD pattern of CCC, indicating the presence of a different Co species (i.e., Co_9_Se_8_) in CNCC that is absent from CCC. The crystal structure models of CoSe, Co_9_Se_8_ and NiSe are shown in Figs. S6a-c, respectively, from which it can be clearly seen that the three substances belong to hexagonal, tetragonal and hexagonal close packed. The doping form of nickel element is shown in Fig. S6d. The introduction of nickel changes the original unit cell structure and the spatial arrangement of some atoms. This structure can theoretically enhance the intrinsic conductivity of the composite system and lead to multiple interface polarization behavior. The lattice distortion caused by the homogeneous doping of Ni elements in Co-ZIF leads to the heterogeneous evolution of Co species during the later selenization treatment, which is considered to be the main reason for the generation of diverse types of selenides in CNCC. Additionally, the carbide intermediates of samples CCC and CNCC are characterized (Fig. S7), confirming that only the characteristic peaks of carbon and reduced metal element existed in the synthesized samples, and no other impurity diffraction peaks are seen in the spectra. Especially, it can be observed from the XRD pattern that the diffraction peaks of the samples CoNi-C@CNFs are relatively close to the positions of the main peaks of both monomeric Co and Ni, which is consistent with the results obtained from the above analysis. The graphitization order degree of carbon-based materials has a considerable influence on the dielectric loss of EMA materials. Therefore, the microstructure and graphitization orderliness of a variety of samples are further investigated using Raman spectroscopy, as shown in Fig. 3b. The characteristic D-band (1360 cm^−1^) and G-band (1580 cm^−1^), which are associated with disordered carbon and crystalline graphitic carbon, respectively, are both readily observed in the spectra of the samples. The ratios of D-band and G-band intensities (*I*_D_/*I*_G_) for Co–C@CNFs and CoNi-C@CNFs are 1.03 and 1.07. The difference between these two values implies some disruption of the crystal structure of the carbon species when some of the Co^2+^ ions in the Co-ZIF structure are replaced by Ni^2+^ ions, resulting in more defects in the carbon structure during the carbonization treatment. The *I*_D_/*I*_G_ values of CCC and CNCC decrease to 0.92 and 0.95, respectively. Compared with Co–C@CNFs and CoNi-C@CNFs, the carbon element of these two selenide composites undergoes a certain degree of structural adjustment and the degree of graphitization has been improved to some extent due to the occurrence of selenization reaction in the later stage. The difference between these two values also indicates that there are more defects in the carbon species of sample CNCC relative to CCC. The five peaks in the sample CNCC with Raman shifts below 1000 cm^−1^ are attributed to the ${\text{A}}_{\text{g}}$, ${\text{E}}_{\text{g}}$, ${\text{F}}_{\text{2g}}^{1}$, ${\text{F}}_{\text{2g}}^{2}$, and ${\text{A}}_{\text{1g}}$ vibrational modes of the Co-Se/Ni-Se bond, which provide the structural fingerprint for the metal selenide [52]. The position of these peaks shifts slightly in the negative direction in CNCC compared to CCC, and the shift in the Raman peak denotes a slight decrease in oscillation frequency, which is connected to heterogeneous interfacial interactions between the various selenide particles. Due to the reduction of the unit cell volume of cobalt selenide due to the introduction of Ni, the spatial arrangement of the selenide species also changes, resulting in rich heterogeneous interfaces between different selenides [53, 54]. The thermochemical processes of PAN fibers and CoNi-ZIF/PAN fibers are investigated using thermogravimetric analyzer and the corresponding thermogravimetric analysis (TGA) curves and derivative thermogravimetric analysis (DTG) curves under an inert atmosphere are shown in Figs. 3c and S8, respectively. At temperatures between 280 and 350 °C and above 400 °C, CoNi-ZIF/PAN fibrous composite exhibit faster mass loss processes. These two mass loss stages of CoNi-ZIF/PAN show similar thermal decomposition processes to pure PAN fibers, corresponding to the stabilization and carbonization processes, respectively. The stabilization stage involves the dehydrogenation, cyclization, and cross-linking of the polyacrylonitrile molecules and results in a mass loss of approximately 15%. In the carbonization stage, the removal of non-carbon light elements, the random breakage of molecular chains, and the structural reorganization of carbon elements occur continuously. This stage ensures the stability of the carbon fiber skeleton and is a necessary process for carbon fiber materials to obtain mechanical strength [55, 56]. In addition, the sample CoNi-ZIF/PAN exhibits two thermal decomposition stages that are different from pure PAN fibers, corresponding to the removal of adsorbed water between the ZIF structural layers (below 250 °C) and the decomposition process of the organic ligands (500–550 °C), which cause a mass loss of 16% and 8%, respectively. The latter process of mass loss overlaps with the carbonization process of pure PAN fibers. After these thermal decomposition processes, the ZIFs loss a large amount of H, O, N, and other elements without destroying the basic structure of the carbon nanosheet framework, which provides the necessary support for the subsequent selenization treatment [57]. Furthermore, the high-temperature carbonization process removes lots of surface hydrophilic functional groups from the fibrous composites, which is conducive to the formation of a strong hydrophobic surface. The surface resistance values of copper strip (Cu strip) and various fibrous membranes are tested with a multi-functional multimeter, and the test demonstration diagram and statistical histogram are shown in Fig. S9a, b, respectively. Before measuring, the copper strip is thoroughly sanded with fine sandpaper to remove the oxide layer on the surface, and then cut into a narrow strip of 2 cm in length and 5 mm in width, and the other fibrous membranes are cut to the same size (Table S1). As demonstrated in Fig. S9b, the copper strip serves as a standard comparison sample possesses the lowest resistance of approximately 1.78 × 10^–3^ Ω. Compared to unmodified CNFs and CCC, the CNCC fibrous composite exhibits the lowest resistance of approximately 2.38 × 10^–2^ Ω, indicating the highest electron transmission efficiency. The excellent electrical conductivity of the fibrous composite membrane stems from its rich internal electron transport network, which favorably enhances a variety of loss capabilities including conduction losses. XPS measurements are carried out to further analyze the surface element state of various samples. The surface element composition and content of various samples are shown in Table S2. As can be observed from the total XPS spectrum in Fig. 3d, the sample CNCC contains the characteristic peaks of C 1s, O 1s, Co 2p, Ni 2p, and Se 3d. The high-resolution XPS spectra in Fig. 3e–i show the valence of each element in the sample CNCC. The high-resolution C 1s spectrum (Fig. 3e) is deconvoluted into three peaks with binding energies of 284.6, 285.7, and 288.4 eV, associated with C–C/C = C, C–O, and C = O bonds, respectively. The characteristic peaks at 286.7 eV in the C 1s spectra of samples Co–C@CNFs and CoNi-C@CNFs are assigned to C = N, while there are no characteristic peaks of nitride species in the C 1s spectra of CCC and CNCC (Fig. S10a). The four diffraction peaks in the N 1s spectra of samples Co–C@CNFs and CoNi-C@CNFs are assigned to graphite N, pyrrole N, M–N, and pyridine N, respectively (Fig. S10b). Specifically, M refers to metal elements, and M–N represents Co–N (Fig. S10c) and Ni–N (Fig. S10d) [58, 59]. In the O 1s spectrum (Fig. 3f), the characteristic peaks around 530.2, 531.3, and 531.8 eV correspond to oxygen species including M–O, Se-O, and surface-adsorbed oxygen, respectively. In the high-resolution spectrum of Fig. 3g, the fitting curve of Co 2p is deconvoluted into six individual characteristic peaks, including four spin–orbit doublets and two satellite peaks. The binding energies of these characteristic peaks are 778.4 and 793.9 eV, 781.5 and 797.1 eV, 785.9 and 802.9 eV, respectively. Specifically, the three pairs of characteristic peaks in the Co 2p spectrum are attributed to Co^3+^, Co^2+^ and satellite peaks, respectively. As shown in Fig. 3h, the fitting of the characteristic peaks in Ni 2p is roughly the same as that of Co 2p, which also contains six individual characteristic peaks, with binding energies of 872.6 and 854.5 eV, 874.6 and 857.1 eV, 880.1 and 860.7 eV, respectively. These three pairs of peaks belong to Ni^2+^, Ni^3+^ and satellite peaks, respectively. In the spectrum of Se 3d (Fig. 3i), the binding energies at 54.2 and 55.1 correspond to Se 2d_5/2_ and Se 2d_3/2_, respectively. In addition, the characteristic peak at 60.8 eV proves the presence of Se-M bond in CNCC, and the Se-O peak at 59.3 eV originates from the trace amount of selenium oxide formed on the sample surface exposed to air [60]. The total XPS spectrum and Fig. S10 show that the samples Co–C@CNFs and CoNi-C@CNFs contain a large amount of N elements, while no obvious diffraction peak of N 1s is observed in CCC or CNCC, and there is no characteristic peak of C-N or C=N in the C 1s spectra. Combined with Table S2 and Raman analysis, it can be inferred that the fibrous membranes via the post-selenization treatment have eliminated residual N element from the initial carbonization process to a large extent. Additionally, the graphitization of carbon elements in fibrous composites has further improved and the structure of the carbon skeleton has become more complete. The hierarchical electron transport networks consisting of highly conductive carbon nanosheets and carbon nanofibers contribute to enhanced conduction loss.

### Electromagnetic Performance and Mechanism

**Fig. 4:**
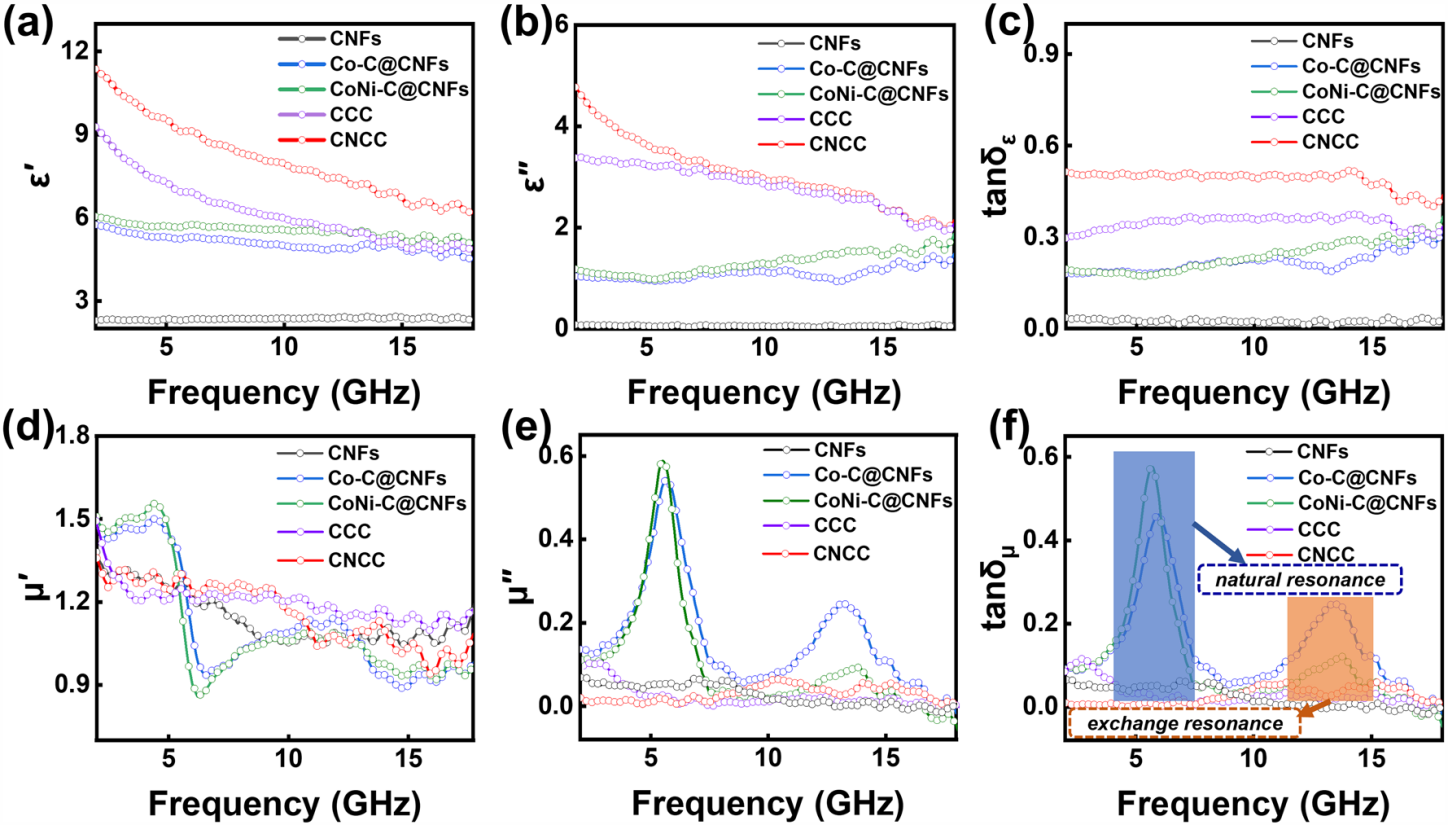
**a** The real part and **b** imaginary part of permittivity, **c** the curves of tanδ_ε_, **d** the real part and **e** imaginary part of permeability, and **f** the curves of tanδ_μ_ of CNFs, Co–C@CNFs, CoNi-C@CNFs, CCC, and CNCC

In order to investigate the factors affecting the EMA performance, the electromagnetic parameters of each sample are analyzed, as illustrated in Fig. 4. In a general way, the complex permittivity (ε_*r*_ = ε′-jε″) and the complex permeability (μ_*r*_ = μ′-jμ″) have a direct influence on EMA performance of absorbers. In detail, the real parts (ε′ and μ′) are commonly used to evaluate the ability to store electromagnetic energy, while the imaginary parts (ε″ and μ″) are used to evaluate the ability to dissipate electromagnetic energy [61–63]. The complex dielectric constants for various samples within the tested frequency range are shown in Fig. 4a, b. Due to the presence of more active sites and higher conductivity in the double transition metal selenides, resulting in an effective dielectric response, the sample CNCC exhibits the most prominent real and imaginary values of the dielectric constant among all the samples. Specifically, the ε′ value of CNCC remains between 6.2 and 11.4 across the whole range, and exhibits a progressive decline as a result of dispersion effects. The ε′ values of other samples are all considerably lower than 9, indicating that the sample CNCC has the strongest ability to store electromagnetic energy [64]. Similarly, CNCC presents higher ε″ value than the other samples in the whole test frequency range, demonstrating that this sample has a strongest electromagnetic wave attenuation ability. In addition, the ε′ and ε″ curves of both samples CCC and CNCC show some fluctuations in the mid-high frequency band, indicating the presence of rich polarization behavior, such as interfacial polarization and dipole polarization, which is conducive to enhance the EMA performance. In contrast, the unmodified CNFs exhibits the smallest ε′ value (< 3) and ε″ value (< 1) among all samples due to absence of effective loss mechanism. Furthermore, the dielectric loss angle tangent $\left( {{\text{tan}}\delta_{{\upvarepsilon }} = \frac{{\varepsilon^{ {\prime \prime }} }}{{\varepsilon  {\prime }}}} \right)$ of CNCC exhibits similar behavior to the ε″ curve, suggesting that multiple polarization relaxation occurs in the sample (Fig. 4c). According to the free electron theory $\left( {\varepsilon ^{{{\prime \prime }}}  \approx \frac{\sigma }{{2\pi \varepsilon _{0} f}}} \right)$, the ε″ value is positively correlated with the permittivity (σ), where ε_0_ represents the permittivity in free space. Obviously, the sample CNCC exhibits the highest conductivity compared to the other samples, which is consistent with the previous analysis results of surface resistance measured by multimeter. The high conductivity of the sample CNCC is attributed to the CNFs frameworks and the large number of CNSs arrays modified on the surface of nanofibers, which in combination with each other can reduce the percolation threshold and construct regional conductive pathways [65]. The presence of metal selenides introduces rich active sites, which further increases the electron transfer efficiency, thereby greatly enhancing the conduction loss of the fibrous composite [66]. The curves of complex permeability and magnetic loss tangent $\left( {\tan \delta _{\mu }  = \frac{{\mu ^{{{\prime \prime }}} }}{{\mu ^{{\prime }} }}} \right)$ for all samples are shown in Fig. 4d–f. The samples Co–C@CNFs and CoNi-C@CNFs generate distinct and violent fluctuations around 5 and 13 GHz, which correspond to free resonance and exchange resonance, respectively. This occurrence is due to the irregular motion of the internal charges of the magnetic components excited by the alternating electromagnetic field, which results in a change in the internal energy of the composites [67]. Generally, the magnetic loss of the absorber in the test frequency range of 2 ~ 18 GHz mainly comes from the natural resonance, exchange resonance and eddy current loss. If the C_0_ $\left( {{\text{C}}_{{\text{0}}}  = \frac{{\mu ^{{{\prime \prime }}} \left( {\mu ^{{\prime }} } \right)^{2} }}{f}} \right)$ values remains constant with frequency, eddy current loss is the primary cause of the magnetic loss in the absorber. On the contrary, it indicates that the magnetic loss is mostly caused by the natural resonance [68]. As shown in Fig. S11, the C_0_ values of the Co–C@CNFs and CoNi-C@CNFs fluctuate very obviously in the regions of 6 and 14 GHz, implying that the main source of magnetic loss for these two samples is not eddy current loss. Except for the sample Co–C@CNFs and CoNi-C@CNFs, the complex permeability curves of other samples maintain a relatively stable trend in the frequency range of 2 ~ 18 GHz without obvious fluctuations, indicating that the contribution of magnetic loss to the EMA performance of samples CCC and CNCC does not account for leading position.

**Fig. 5:**
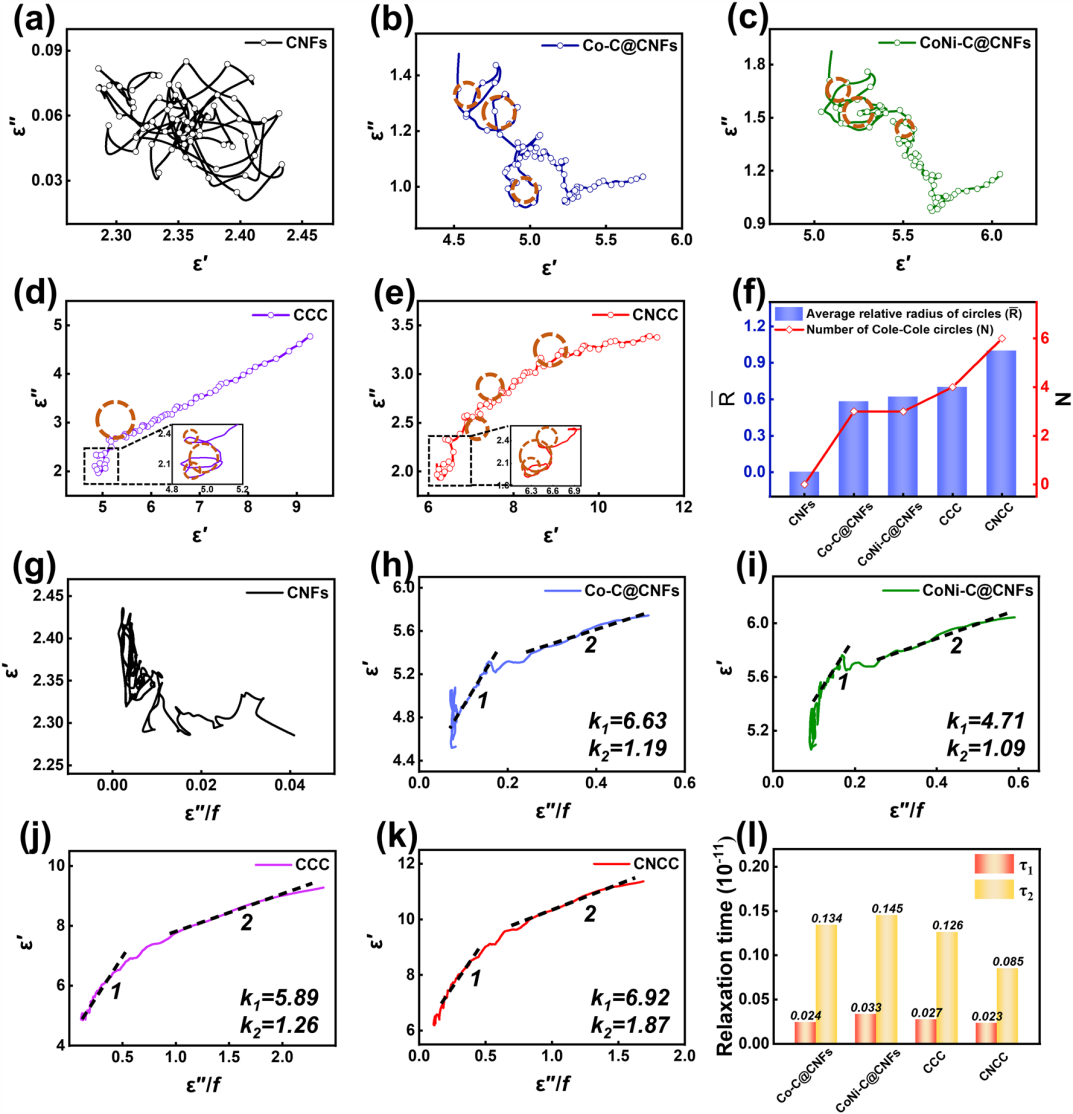
The Cole–Cole plots of **a** unmodified CNFs, **b** Co–C@CNFs, **c** CoNi-C@CNFs, **d** CCC, and **e** CNCC, **f** statistics on the number and average relative radius (with respect to CNCC) of circles in the Cole–Cole curve for all samples, the relationship between ε′ and ε″/*f* values of **g** unmodified CNFs, **h** Co–C@CNFs, **i** CoNi-C@CNFs, **j** CCC, **k** CNCC, and **l** statistics on relaxation times for all samples

To further reveal the polarization relaxation mechanism, the Cole–Cole curves of all samples are plotted in Figs. 5a–e, respectively. Based on the support of the Debye relaxation theory, the relationship between ε′ and ε″ can be revealed by the following equations [69–71]:

3 $$ \varepsilon^{ {\prime }} { = }\varepsilon_{\infty } + \frac{{\varepsilon_{s} - \varepsilon_{\infty } }}{{1 + \left( {2\pi f} \right)^{2} \tau^{2} }} $$

4 $$ \varepsilon^{{ {\prime \prime }}} { = }\frac{{2\pi f\tau \left( {\varepsilon_{s} - \varepsilon_{\infty } } \right)}}{{1 + \left( {2\pi f} \right)^{2} \tau^{2} }} $$

5 $$ \left( {\varepsilon^{ {\prime }} - \frac{{\varepsilon_{s} - \varepsilon_{\infty } }}{2}} \right)^{2} + \left( {\varepsilon^{{ {\prime \prime }}} } \right)^{2} { = }\left( {\frac{{\varepsilon_{s} - \varepsilon_{\infty } }}{2}} \right)^{2} $$

where* ε*_s_ $\left( {{\upvarepsilon }_{s} = \mathop {\lim }\limits_{{{\upomega } \to 0}} {\upvarepsilon }_{r} } \right)$ and* ε*_∞_ $\left( {{\upvarepsilon }_{\infty } = \mathop {\lim }\limits_{{{\upomega } \to \infty }} {\upvarepsilon }_{r} } \right)$ are the static and optimal dielectric constants, and *τ* and* ω* ($\upomega =2\uppi f$) represent the polarization relaxation time and angular frequency, respectively. In general, the Cole–Cole curve can be used to represent the polarization effect of an EMA materials, where each semicircle represents a relaxation process. The absence of significant semicircles in the Cole–Cole curve for unmodified CNFs proves that there is no considerable polarization relaxation behavior in this sample (Fig. 5a). The polarization relaxation behaviors of the fibrous composites have been significantly improved after surface modification with ZIF nanoarrays, carbonization, and selenization (Fig. 5b–d). Especially, multiple relaxation semicircles are presented in Fig. 5e, which indicates the rich relaxation behavior in the CNCC fibrous composite. Compared with the Cole–Cole curves of other samples (Fig. 5f), both the number and size of the semicircles of the sample CNCC are dominant, which is attributed to the dipole polarization of defect-induced charge formation and the interfacial polarization between the multicomponent heterogeneous interfaces within the composite. According to Eq. (4), the polarization relaxation time can be expressed as follows:

6 $$ \varepsilon^{ {\prime }} { = }\frac{1}{2\pi \tau }\frac{{\varepsilon^{{ {\prime \prime }}} }}{f} + \varepsilon_{\infty } $$

According to the equation, when polarization loss occurs in dielectric loss, the relationship between* ε*′ and* ε*″/*f* is a straight line with a slope of 1/2π*τ*, from which the relaxation time *τ* for polarization relaxation can be calculated. As shown in Fig. 5g, the absence of the part of the curve corresponding to the straight line in the unmodified CNFs proves that there is no polarization relaxation behavior in this sample. The* ε*′ −*ε*″/*f* curves for the other composites all contained straight-line portions of two segments (Figs. 5h–k), i.e., corresponding to two relaxation times. As shown in Fig. 5l, the relaxation times for various samples are 0.024 and 0.134 for Co–C@CNFs, 0.033 and 0.145 for CoNi-C@CNFs, 0.027 and 0.126 for CCC, and 0.023 and 0.085 for CNCC, respectively. Obviously, the polarization relaxation times of these samples are not unique, which may be due to the presence of multiple polarization relaxation processes in the composites. This phenomenon proves that different polarization relaxation processes have individual relaxation times and contribute differently to the attenuated electromagnetic wave energy [72]. In this case, polarization relaxation mainly consists of interfacial polarization and dipole polarization. Specifically, the rich heterogeneous interfaces between different selenide nanoparticles under the action of an applied electromagnetic field promotes interfacial polarization, and an abundance of defects in the carbon fiber frameworks and carbon nanosheet arrays obtained via the carbonization process induces dipole polarization. It can be inferred that the sample CNCC with hierarchical multilevel heterostructure has the highest dielectric loss capability, which is mainly due to the abundant micro-conducting networks, local defects, and heterogeneous interfaces within the composites. In order to achieve excellent EMA performance, a qualified absorber should satisfy both appropriate impedance matching (Z) and outstanding attenuation constant (α). Impedance matching is to design the input impedance of the absorber to be consistent with the free space impedance to minimize electromagnetic wave reflection. Appropriate impedance matching means that more electromagnetic waves enter the interior of the absorber instead of being reflected on its surface, which is a prerequisite for efficient attenuation of electromagnetic waves. The impedance matching characteristic can be described by the following equation [73]:

7 $$ Z = \left| {\frac{{Z_{in} }}{{Z_{0} }}} \right| $$

The closer the value of* Z* is to 1, the better the impedance match of the EMA material. The value of* Z* is related to the frequency of the electromagnetic wave and the thickness of the absorber. The matching thickness (*t*_*m*_) versus frequency can be simulated by the quarter-wavelength (*λ*/4) model as follows [74, 75]:

8 $$  {\text{t}}_{{\text{m}}}  = \frac{{n\lambda }}{4} = \frac{{nc}}{{4f_{m} \sqrt {\left| {\varepsilon _{r} \mu _{r} } \right|} }}\left( {n = 1,3,5 \ldots } \right) $$

where *λ* is the wavelength of the electromagnetic waves, *c* is the propagation velocity of electromagnetic waves in a vacuum, and |ε_*r*_| and |μ_*r*_| represent the moduli of ε_*r*_ and μ_*r*_, respectively. Based on the *λ*/4 model, when the matching thickness is equal to the wavelength of the medium multiplied by an odd number, the incident waves and reflected waves at the front and rear interfaces of the absorber medium will interfere and cancel, resulting in consumption. Especially, complete interference cancellation occurs when the two electromagnetic waves have the same amplitude. The dot symbols (*●*) in Fig. S12 (corresponding to the thickness of the RL_min_ value) are almost always above the λ/4 curve (*t*_*m*_), i.e., indicating that the matching thickness value corresponding to RL_min_ closely matches the simulated curve drawn according to Eq. (8). Apparently, the CNCC fibrous composite satisfy the quarter-wavelength model, indicating that the loss mode of this absorber is interferometric [76, 77]. As shown in Fig. S13, the percentage of area occupied by the specific impedance matching interval (0.8 < Z < 1.2) for sample CNCC is the highest of all samples at 14.3%, much higher than unmodified CNFs (9.38%), Co–C@CNFs (10.4%), CoNi-C@CNFs (9.76%) and CCC (11.2%). It is obvious that CNCC exhibits the most excellent impedance matching condition compared with other samples. The ideal impedance match can be attributed to the interconnected conductive network structure resulting from the combination of selenide particles, carbon nanosheet arrays, and carbon nanofibers, which provides multiple channels for electron transport and migration. Another critical factor in achieving efficient EMA performance is the attenuation constant (α), which reflects the ability of the electromagnetic wave to be attenuated inside the absorber. The α value can be calculated by the following equation:

9 $$ \alpha = \frac{\sqrt 2 \pi f}{c}\sqrt {\left( {\mu^{{ {\prime \prime }}} \varepsilon^{ {\prime \prime }} - \mu^{ {\prime }} \varepsilon^{ {\prime }} } \right) + \sqrt {\left( {\mu^{{ {\prime \prime }}} \varepsilon^{ {\prime \prime }} - \mu^{ {\prime }} \varepsilon^{ {\prime }} } \right)^{2} + \left( {\mu^{ {\prime }} \varepsilon^{ {\prime \prime }} - \mu^{{ {\prime \prime }}} \varepsilon^{ {\prime }} } \right)^{2} } } $$

As shown in Fig. S14, the α value of CNCC is much higher than the other samples throughout the frequency range, indicating the strongest dissipation performance of electromagnetic waves. In addition, the attenuation constant curve of sample CNCC shows a monotonic increase with increasing frequency, indicating that electromagnetic waves at high frequencies are more easily to attenuate in the absorber. It should be emphasized that the high α value and eximious Z value are somewhat mutually exclusive, making it challenging to meet both objectives concurrently for traditional EMA materials [78]. Here, an optimal balance between the two can be achieved in CNCC fibrous composite through rational composition modulation and precise structural design. Therefore, the excellent impedance matching and extraordinary attenuation capability are the keys to efficient EMA performance of CNCC fibrous composite.

**Fig. 6:**
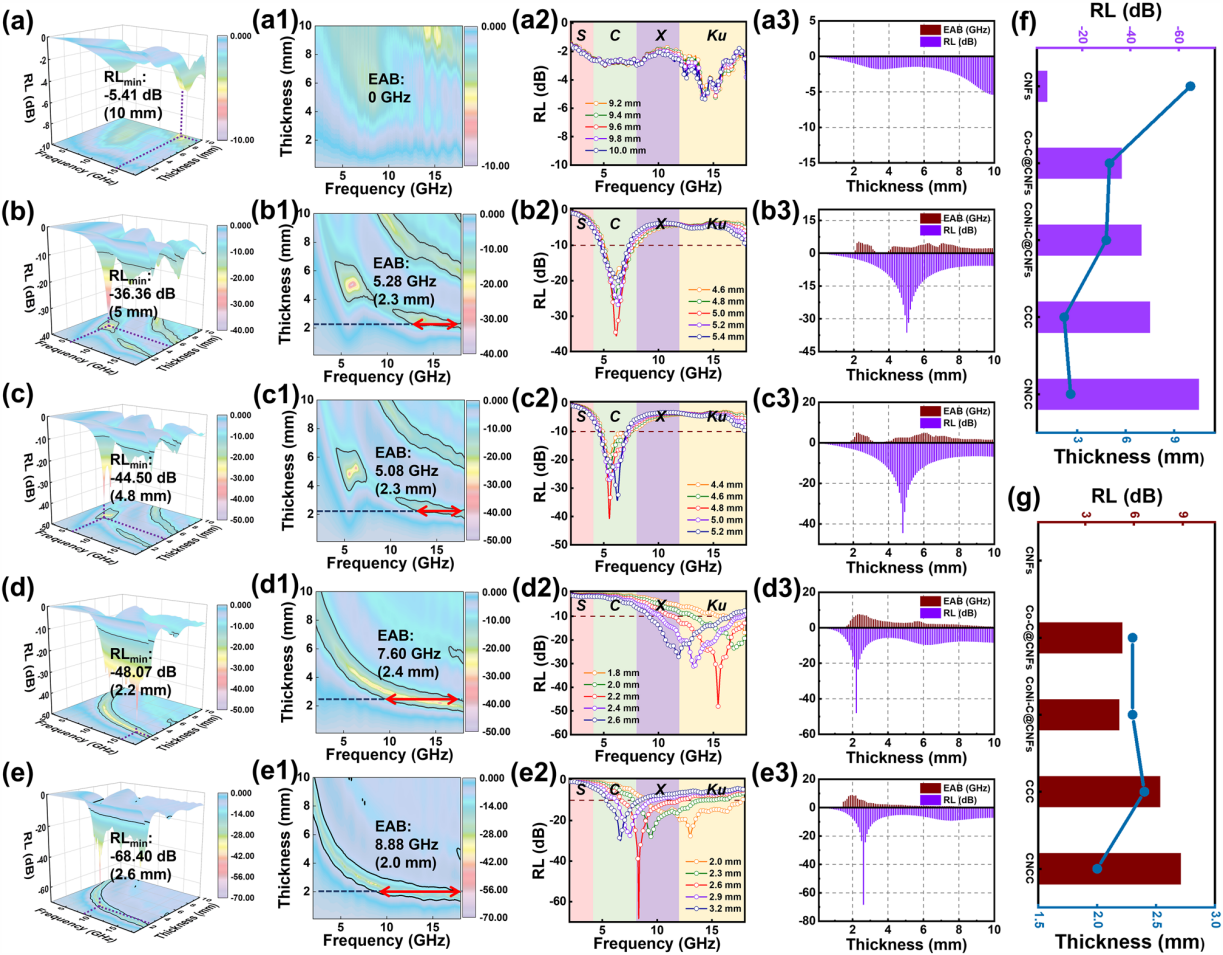
The reflection loss values and effective absorption bandwidth of all samples varying with thickness and frequency: **a** unmodified CNFs, **b** Co–C@CNFs, **c** CoNi-C@CNFs, **d** CCC, **e** CNCC, and the comparison plots of **f** RL and **g** EAB corresponding to various matching thicknesses

In order to visually compare the EMA properties of various composites, the reflection loss values and effective absorption bandwidth of all samples varying with thickness and frequency are investigated in Fig. 6. Obviously, the unmodified CNFs exhibits the worst EMA performance with a minimum RL value of only − 5.41 dB among these samples, implying that no effective absorption bandwidth exists for this sample in the tested frequency range. The performance of EMA is significantly enhanced with the metal–carbon nanosheet arrays modified on the surface of CNFs. The RL_min_ values of − 36.36 and − 44.50 dB and maximum EAB values of 5.28 and 5.08 GHz are achieved for Co–C@CNFs and CoNi-C@CNFs with corresponding matched thicknesses, respectively (Fig. 6b–c). The presence of the nanosheet arrays not only promotes multiple reflection and scattering of electromagnetic waves inside the composites, but also increases the contact area between the nanofibers, thus enabling the fibrous composites to build sufficient conductive channels in the paraffin matrix even at low fillings, optimizing a variety of loss mechanisms including conduction losses. The absorption properties of the fibrous composites are further enhanced via the selenization modification. The sample CCC achieves an RL_min_ value of − 48.07 dB (2.2 mm) and an EAB value of 7.6 GHz (2.4 mm), which are respectively increased by 32% and 43% compared with Co–C@CNFs, respectively (Fig. 6d). Most significantly, the sample CNCC in Fig. 6e exhibits the most satisfactory EMA performance, with a minimum RL value of − 68.40 dB at 2.6 mm and a maximum EAB value of 8.88 GHz at a thin thickness of 2.0 mm (covering 75% of the X and all Ku bands). Especially, by adjusting the thickness from 1.5 to 5 mm, the sample CNCC obtains an effective absorption bandwidth of 14.72 GHz, achieving full coverage of the entire C, X, and Ku bands, which occupies 92% of the overall tested frequency range (Fig. S15a). In addition, the sample CNCC still achieves an RL_min_ value close to -50 dB and an EAB of 6.08 GHz at an ultra-thin thickness of 1.7 mm (Fig. S15b).

To further reveal the EMA performance of CNCC fibrous composite, performance data for other typical 1D composites and MOF-derived composites are presented in Tables S3 and S4, respectively, with corresponding intuitive comparison plots (Fig. S16). The CNCC fibrous composite prepared in this work achieves an ultra-high specific RL of − 1368 dB (RL_min_/filling load) and an ultra-wide specific EAB of 177.6 GHz (EAB/filling load) at thinner thicknesses, exhibiting ultra-efficient EMA performance. Compared to other 1D fiber composites, CCC and CNCC fibrous composites with rich multi-level heterostructures exhibit significantly thinner matched thicknesses (Figs. S16a–b). Compared to the powdery MOF-derived composites, the absorbers prepared in this work have an obvious outstanding advantage in terms of filler loading, with an order of magnitude difference in the specific RL and specific EAB values (Figs. S16d–e). Taken as a whole, these substantial benefits of low filling load, thin matching thickness, wide effective absorption bandwidth, and excellent absorption performance are all present in the CNCC fibrous composite with MOF-derived hierarchical heterostructures prepared in this work (Figs. S16c, g). These impressive results demonstrate that by rationally designing the hierarchical structure of MOF-derived materials and optimizing the loss mechanisms and synergistic effects of multiple components, the absorption parameters of fibrous composites can be tuned and improved, resulting in satisfactory EMA performance.

**Scheme 1:**
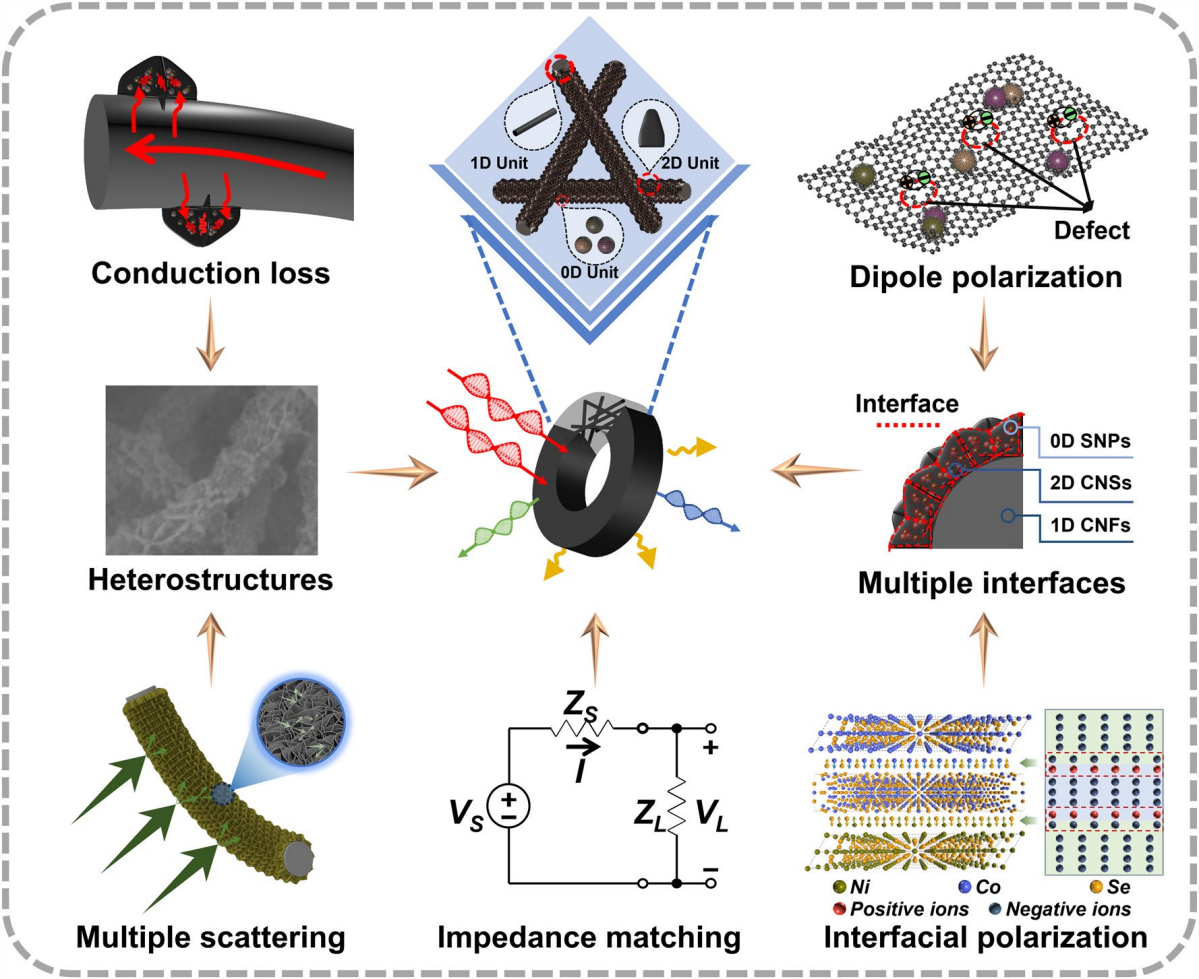
The electromagnetic protection mechanism of CNCC composite fabric

Scheme 1 illustrates the EMA mechanism of CNCC electromagnetic protective fabric. Firstly, the petal-like 2D nanosheet structure modified on the nanofibrous framework introduces abundant void space into the composite system, allowing more electromagnetic waves to penetrate the surface and enter the interior of the composite, thereby optimizing the impedance matching. Secondly, due to the presence of hierarchical carbon species, there are differences in the crystal states and electron transport efficiencies of different carbon materials, the carriers will be trapped by the interface during the migration process, resulting in the accumulation and heterogeneous distribution of charges around the interface region, which can generate abundant interfacial polarization. In addition, the abundant defects in the carbon nanoarrays can act as polarization centers, leading to dipolar relaxation. In brief, the abundance of heterogeneous interfaces between different selenide nanoparticles and between selenide and carbon matrix promote interfacial polarization, and the rich defects on the highly porous MOF-derived carbon nanosheets act as active sites to induce dipole polarization, leading to the composite with high effective dielectric constants, which is positive for improving dielectric loss. Furthermore, the carbon nanosheet arrays with high specific surface area derived from MOF precursors facilitate multiple reflection and scattering of electromagnetic waves, extending the propagation distance of the incident waves, and increasing the probability of absorption and dissipation. Finally, the carbon nanofiber frameworks and carbon nanosheet arrays are interconnected to form abundant regional or global conductive networks, which can enhance the conduction loss. Specifically, under the influence of an external electromagnetic field, excited electrons migrate directionally along the carbon fiber skeletons and leap into the adjacent carbon nanosheet arrays, resulting in microcurrents that convert electromagnetic energy into thermal energy [79, 80]. As a result, under the synergistic effect of multiple electromagnetic attenuation mechanisms, the CNCC electromagnetic protective fabric with hierarchical heterostructures have obtained excellent EMA performance, and its outstanding advantages lie in ultra-low filling load, ultra-thin matching thickness, and ultra-wide effective absorption bandwidth.

### Multifunctional Properties

**Fig. 7:**
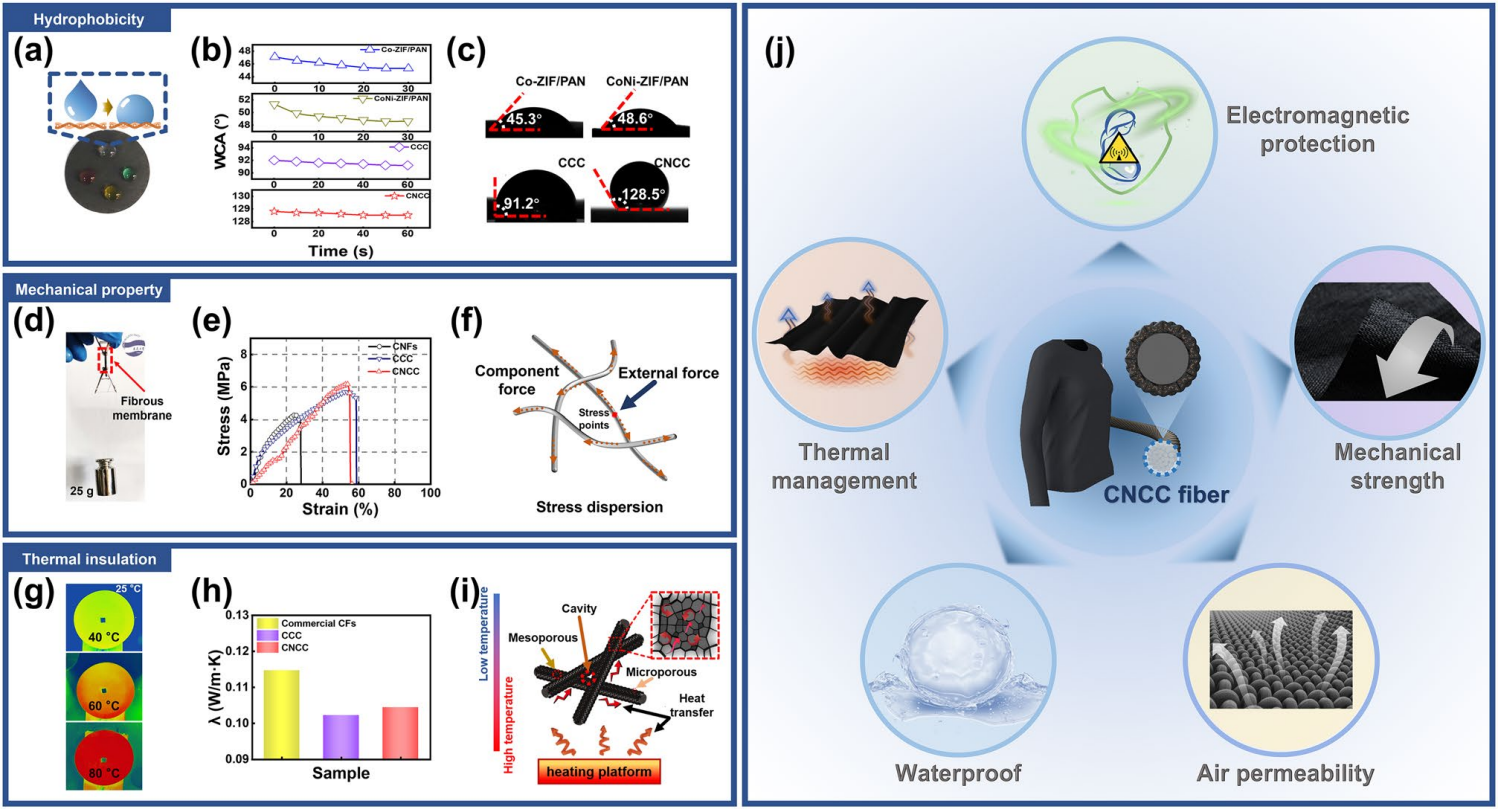
Hydrophobicity: **a** digital image of CNCC fibrous membrane surface dripped with colored droplets, **b** water contact angle test values of various fibrous membranes that vary with time, **c** final fixation values water contact angle values for various fibrous membranes; Mechanical property: **d** the digital image after dropping a 25 g weight under the CNCC fibrous membrane, **e** the stress–strain curves of various fibrous membranes, **f** schematic diagram of the stress dispersion mechanism inside the fibrous membrane; Thermal insulation: **g** infrared thermal pictures of CNCC fibrous membrane on heating platform at different temperatures, **h** statistics of thermal conductivity of various fibrous membranes at 300 K, **i** schematic illustration of thermal insulation mechanism of CNCC fibrous membrane, **j** the application scene design of CNCC multifunctional nanofibrous composite membrane

In addition to the excellent electromagnetic protective function, the CNCC composite fabric also exhibits excellent multi-functional characteristics. The CNCC fibrous membrane displays exceptional hydrophobicity as a result of the elimination of an abundance of hydrophilic functional groups during pyrolysis process and the lowering of the water–solid contact area by the high roughness surface formed by the nano-petals assembly (Fig. 7a). The results of water contact angle tests for various membranes are shown in Fig. 7b, c, with the most hydrophobic CNCC fibrous membrane measuring approximately 128.5°, significantly higher than the precursor fibrous membranes Co-ZIF/PAN (45.3°), CoNi-ZIF/PAN (48.6°) and the monometallic MOF derivative CCC (91.2°) [81]. The strongly hydrophobic surface endows the CNCC composite membrane with excellent adaptability to high-humidity environments such as underwater, foggy weather, and wet skin surfaces. In addition, a simple test reveals that the CNCC fibrous membrane also has excellent mechanical strength, as demonstrated by the fact that the membrane can suspend a 25 g weight at the bottom end without fracture (Fig. 7d). The tensile strength of nanofibrous membranes is of great significance for the evaluation of wear resistance of fabric-based wearable electronics. The mechanical properties of fibrous membranes such as unmodified CNFs, CCC, and CNCC are tested, and the stress–strain curves are shown in Fig. 7e. All stress values are calculated from the cross-sectional area of the fibrous membrane determined by width × thickness (Table S5). The average tensile strength and elongation at break for unmodified CNFs, CCC and CNCC are 4.35 MPa and 27.8%, 5.68 MPa and 59.3%, and 6.16 MPa and 55.7%, respectively. The macroscopic mechanical strength of the fibrous membrane is due to the rapid stress response mechanism of the interconnected network of carbon fibers inside the membrane. When a single fiber is exposed to an external force, the stress can quickly be dispersed to other neighboring fibers, preventing the overall structure of the membrane from being readily broken (Fig. 7f). Moreover, the nano-sheet arrays on the surface of the carbon fiber frameworks allow the adjacent fibers to “interlock” as in a “mortise-tenon connection”, increasing the friction between fibers, improving the interfacial bonding, reducing the relative displacement between stress points, and forming a resilient framework. The interconnected network framework within the fibrous membrane is the structural basis for its macroscopic mechanical stability. Furthermore, the CNCC fibrous composite membrane also exhibits certain thermal management performance. The infrared thermal picture clearly demonstrates a large temperature differential between the membrane surface and the heating platform, as illustrated in Fig. 7g. The CNCC fibrous membrane consistently maintains a temperature close to the ambient (25 °C) as the temperature of heating platform steadily rises to 40, 60, and 80 °C. The thermal conductivity of various fibrous membranes at 300 K is calculated using a portable thermal conductivity meter, as shown in Fig. 7h. The thermal conductivity of CCC and CNCC composite membranes is much lower than that of commercial carbon fiber membranes (0.1147 W m^−1^ K^−1^), which are 0.1022 and 0.1044 W m^−1^ K^−1^, respectively, proving that the composite fabrics achieve relatively good thermal insulation properties. The weak interfacial bonding between fabric fibers with unmodified hierarchical nanoarrays on the surface can become the entrance for thermal failure, destroying the thermal resistance of the fibrous membrane, and resulting in poor thermal insulation ability. It is worth noting that the thermal conductivity of CNCC is slightly higher than that of CCC, which is due to its higher dielectric constant and denser nanosheet arrays on the surface of nanofibers [82]. The temperature difference curves between the upper and lower surfaces of various fiber membranes on the surface of the thermal conductivity test bench within 0–0.09 s are statistically shown in Fig. S17, which also proves that the temperature transfer rate perpendicular to the surface of the CNCC composite membrane is slightly slower than that of conventional carbon fiber fabric within the same time period. The thermal insulation capability of the CNCC composite membrane stems from the high porosity inside the 0D@2D@1D structures and the large amount of air inside the cavity [83, 84]. N_2_ adsorption–desorption and pore measurement are used to analyze the pore structure and size distribution of CNCC fibrous composite membrane with BET specific surface areas (SSAs) and BJH (Barret–Joyner–Halenda) pore size distributions in Fig. S18. The corresponding curves show typical type IV isotherm characteristics, indicating that the fibrous membranes have an abundant mesoporous structure. The pore size distribution curve of the sample CNCC in the inset shows a multi-level pore distribution, confirming the coexistence of internal micropores and mesopores. Due to the introduction of the petal-like ZIF structure, the fibrous composite membrane obtains a large SSA (289.8 m^2^ g^−1^) and abundant pores (0.343 cm^3^ g ^−1^), which significantly reduces the overall fibrous membrane density. In addition, the high porosity inside the fibrous composite membrane and the presence of air in the pores, a poor thermal conductor, can significantly reduce heat transfer and thermal radiation [85]. Figure 7i schematically explains the thermal management mechanism of the CNCC composite fabric. The large cavities formed between the fiber bundles, the mesoporous structure composed of nanosheet arrays, and the microporous structure inside the nanosheets and fibers constitute the intricate hierarchical pore network inside the composite membrane, which effectively hinders the heat conduction between the upper and lower surfaces of the composite fabric. The thermal insulation capability of fibrous composite membrane enables it to avoid infrared radiation detection to a certain extent as well as shield electronic devices from the damaging caused by high temperatures. The CNCC nanofibrous membrane with multifunctional properties such as flexibility, air permeability, waterproof, thermal management, and electromagnetic protection have broad application prospects in the fields of national defense and people’s livelihood (Fig. 7j).

## Conclusions

In summary, we have developed a facile method to fabricate Co_x_Se_y_/NiSe@CNSs@CNFs fibrous composite with hierarchical heterostructures via the targeted induction and vapor-phase selenization process. This unique structure uses graphitized carbon fibers as “branches”, MOF-derived carbon nanosheets as “petals”, and metal selenide nanoparticles as “stamens”, simultaneously realizing abondance of hierarchical conductive networks, rich heterogeneous interfaces, and sufficient space clearance. Due to the synergistic effect of multiple loss mechanisms, the CNCC composite fabric exhibits extremely superior electromagnetic wave absorption capacity at an ultra-low filling load of only 5 wt%. Its RL_min_ value reaches -68.40 dB at 2.6 mm, and an EAB of 8.88 GHz is obtained at a thin thickness of 2.0 mm, which can cover more than half of the test frequency range. In addition, the sophisticated “one for all” structure brings a variety of multifunctional properties such as flexibility, hydrophobicity, mechanical stability, and thermal insulation to the CNCC electromagnetic protective membrane. This work provides some guidance for the precise design and construction of MOF-derived fibrous composites with hierarchical structures for multifunctional protection applications.

### Supplementary Information

Below is the link to the electronic supplementary material.

Supplementary file1 (PDF 2171 KB)
