# Supplementary material for: Integration of Multiple Heterointerfaces in a Hierarchical 0D@2D@1D Structure for Lightweight, Flexible, and Hydrophobic Multifunctional Electromagnetic Protective Fabrics
Source: Nanomicro Lett. 2023 Aug 25;15:204. doi: 10.1007/s40820-023-01179-2 (PMC10457279; doi:10.1007/s40820-023-01179-2)
Supplement: Supplementary file 1 — Supplementary file1 (PDF 2171 KB) [file 40820_2023_1179_MOESM1_ESM.pdf]

Supporting Information for

Integration of Multiple Heterointerfaces in a Hierarchical 0D@2D@1D Structure for Lightweight, Flexible, and Hydrophobic Multifunctional Electromagnetic Protective Fabrics

Shuo Zhang[1, #], Xuehua Liu[1, #], Chenyu Jia[1], Zhengshuo Sun[1], Haowen Jiang[1], Zirui Jia[2, *], Guanglei Wu[1, *]

[1] Institute of Materials for Energy and Environment, State Key Laboratory of Bio-fibers and Eco-textiles, College of Materials Science and Engineering, Qingdao University, Qingdao 266071, P. R. China

[2] College of Chemistry and Chemical Engineering, Qingdao University, Qingdao 266071, P. R. China

# Zirui Jia and Guanglei Wu contributed equally to this work.

*Corresponding authors. E-mail: jiazirui@qdu.edu.cn (Z. Jia), wuguanglei@qdu.edu.cn/wuguanglei@mail.xjtu.edu.cn (G. Wu)

Supplementary Figures

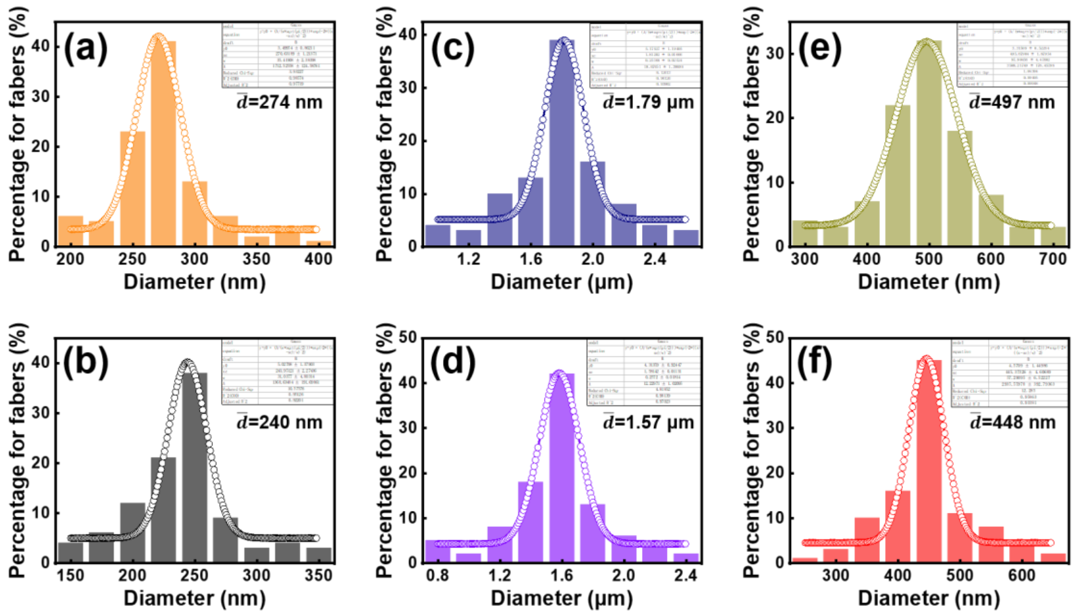

Fig. S1 Statistical histogram of diameter of (**a**) PAN NFs, (**b**) CNFs, (**c**) Co-ZIF/PAN, (**d**) CCC, (**e**) CoNi-ZIF/PAN, and (**f**) CNCC fibrous composites

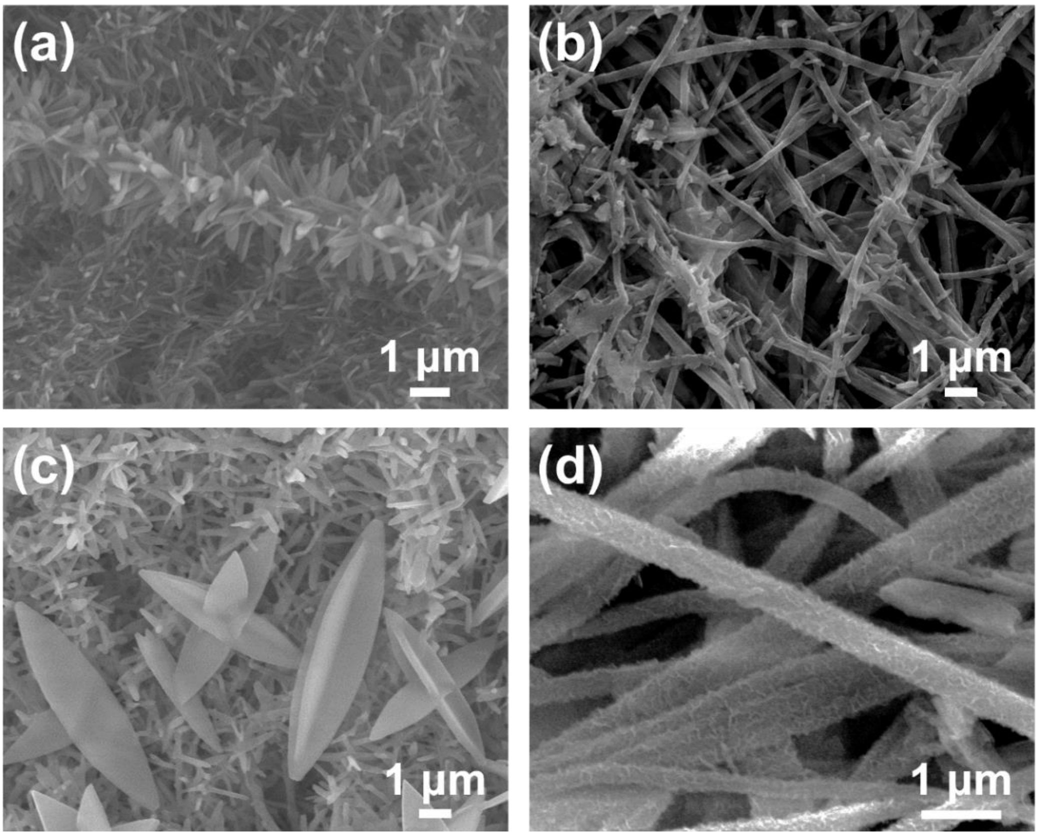

Fig. S2 SEM images of (**a**) Co-ZIF/PAN (Co^{2+}:2-MI=1:8), (**b**) Co-ZIF/PAN (Co^{2+}:2-MI=1:0), (**c**) Co-ZIF/PAN (Co^{2+}:2-MI=1:16), and (**d**) CoNi-ZIF/PAN

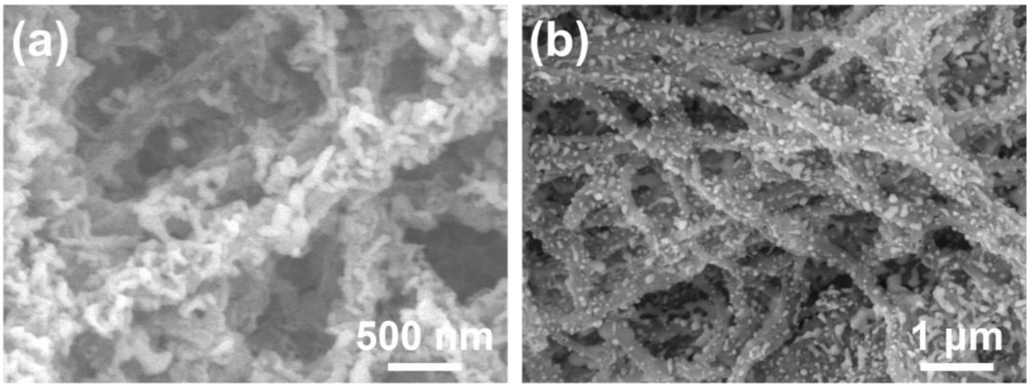

Fig. S3 SEM images of (**a**) Co-ZIF/PAN and (**b**) CoNi-ZIF/PAN directly selenized without pre-carbonization

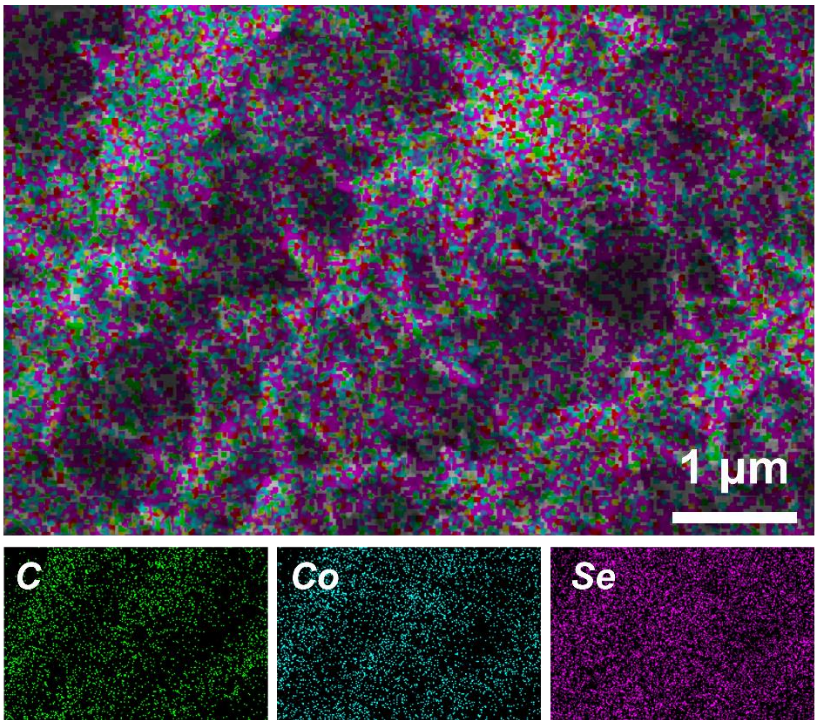

Fig. S4 EDS mapping of sample CCC

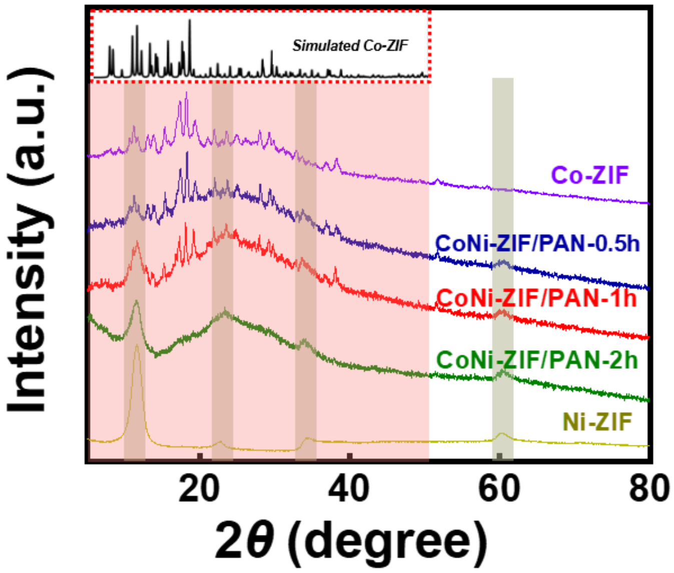

Fig. S5 XRD patterns of single component Co-ZIF, Ni-ZIF and CoNi-ZIF/PAN treated with different sonication times

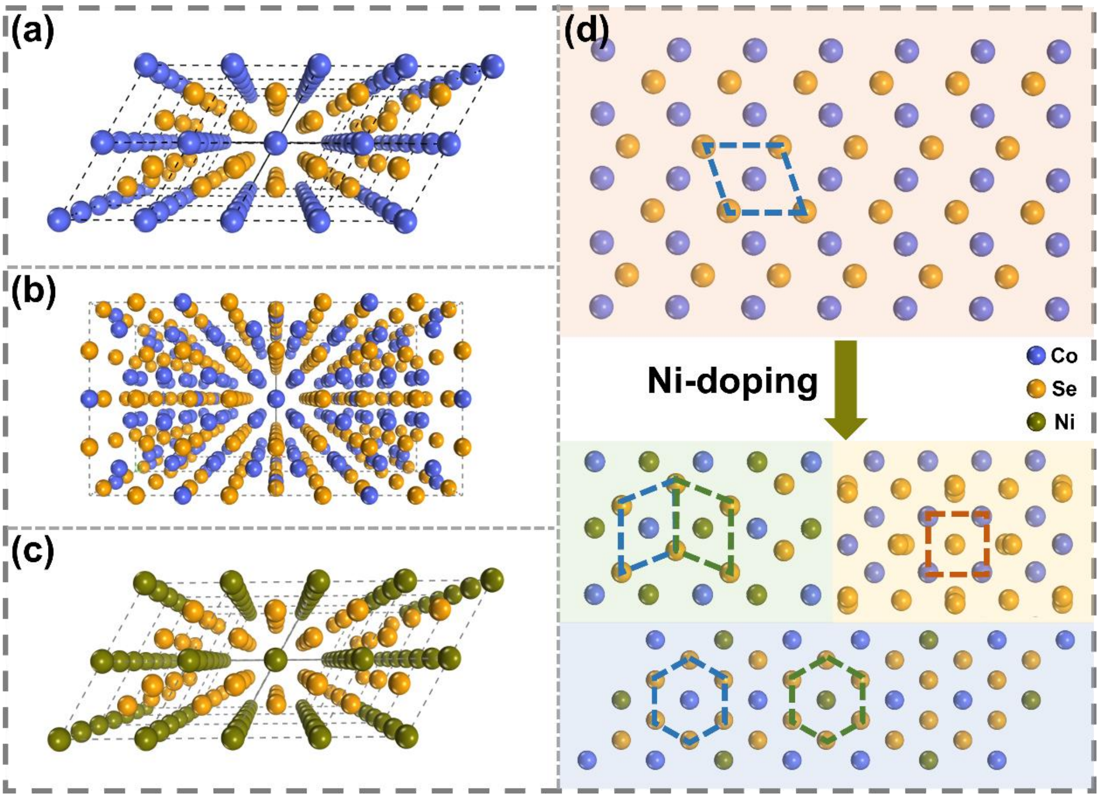

Fig. S6 The crystal structure models of (**a**) hexagonal CoSe, (**b**) tetragonal Co_9Se_8, (**c**) hexagonal NiSe, and (**d**) schematic diagram of the effect of nickel introduction on material transformation

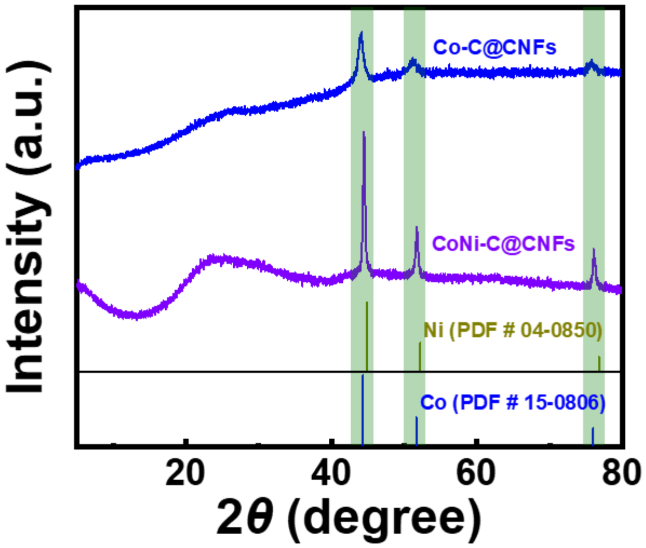

Fig. S7 XRD patterns of Co-C@CNFs and CoNi-C@CNFs

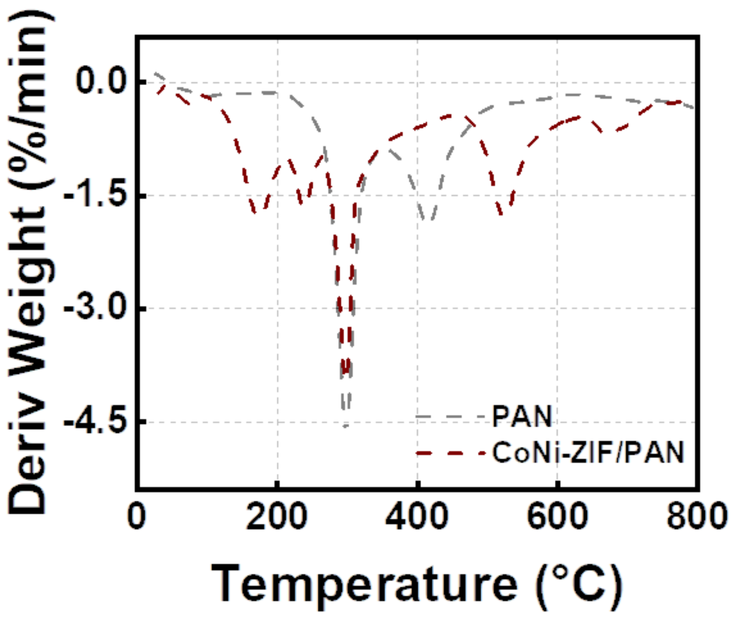

Fig. S8 DTG curves of PAN and CoNi-ZIF/PAN

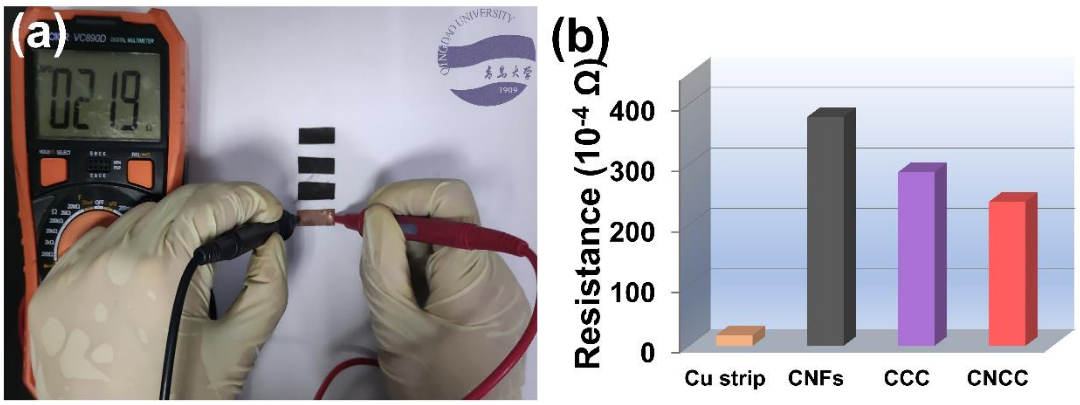

Fig. S9 (**a**) Schematic diagram of surface resistance test of copper strip and various fibrous membranes (the figure shows the test data of copper strip with unpolished surface), (**b**) the statistical histogram of surface resistance test values of all samples

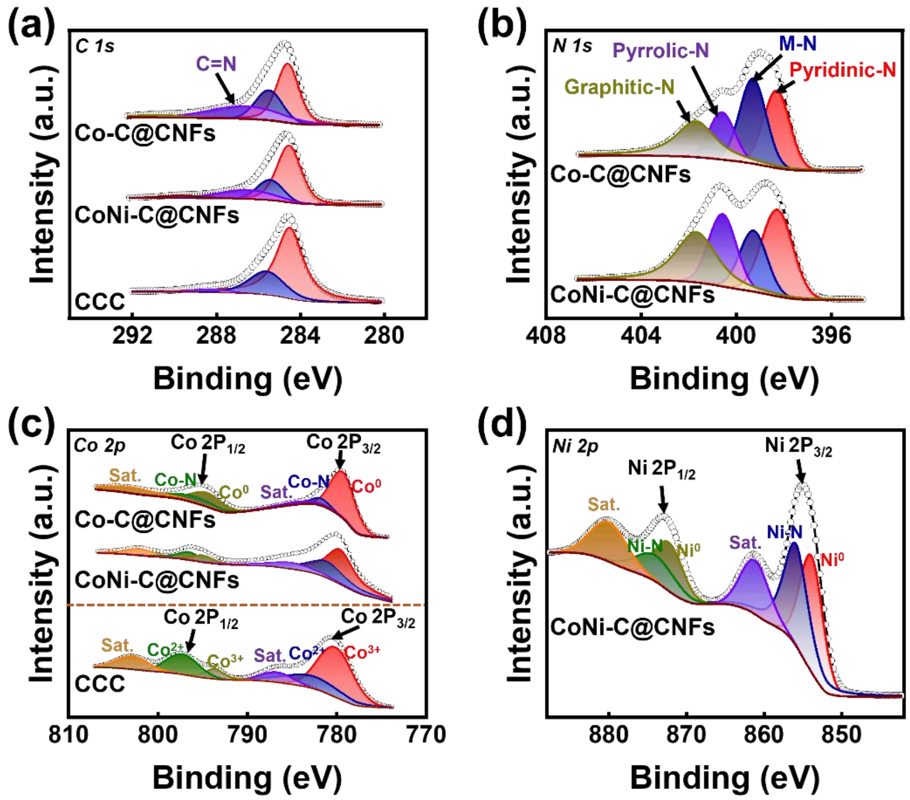

Fig. S10 High-resolution XPS patterns of samples Co-C@CNFs, CoNi-C@CNFs and CCC: (**a**) C 1s, (**b**) N 1s, (**c**) Co 2p and (**d**) Ni 2p

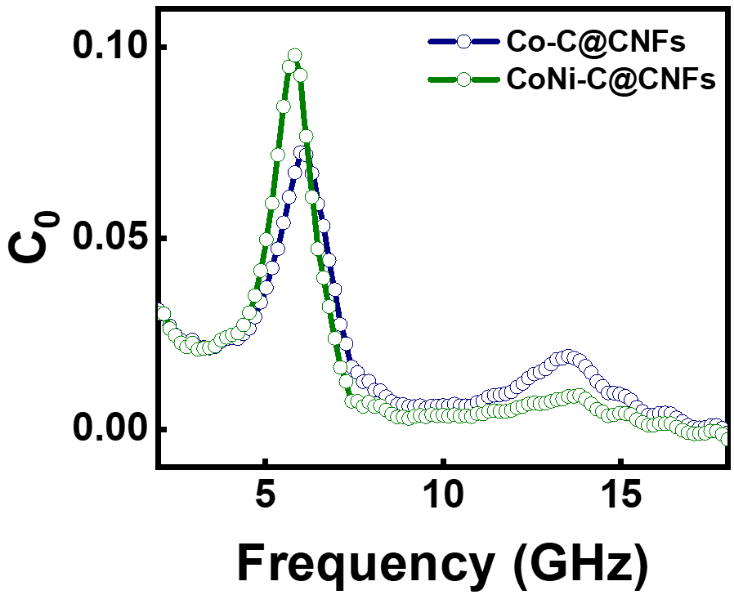

Fig. S11 The C_0 curves of Co-C@CNFs and CoNi-C@CNFs

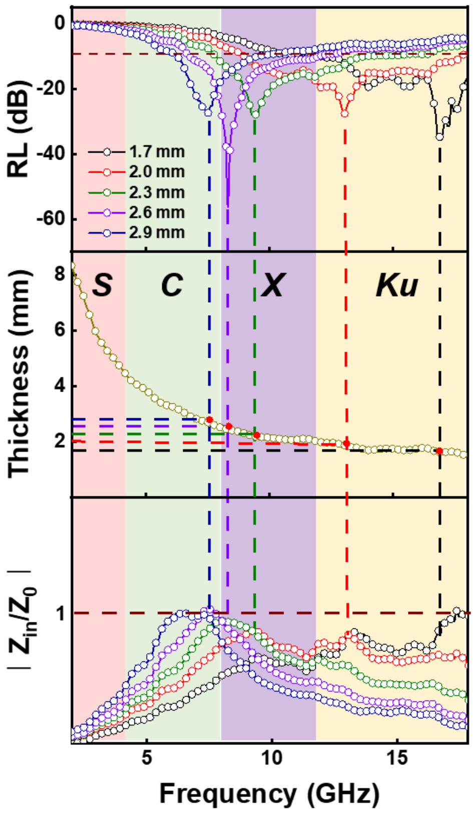

Fig. S12 The dependence plot of $\lambda/4$ matching thickness vs RL peak vs $|Z_{in}/Z_0|$ of the sample CNCC

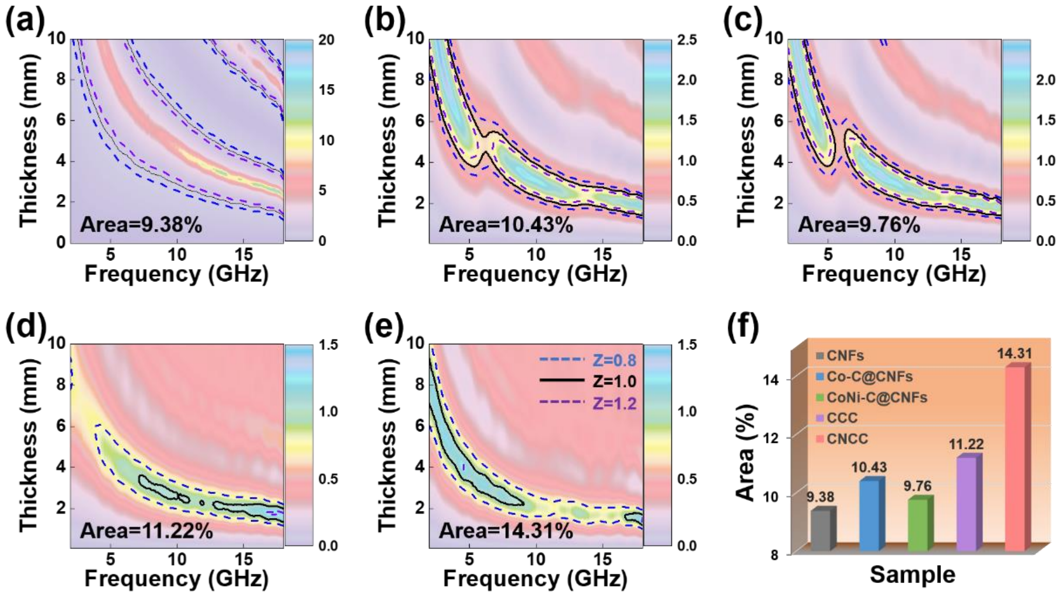

Fig. S13 Contour map of the values of Z of (**a**) CNFs, (**b**) Co-C@ CNFs, (**c**) CoNi-C@ CNFs, (**d**) CCC, (**e**) CNCC, and (**f**) the percentage of area occupied by the specific impedance matching interval (0.8 < Z < 1.2)

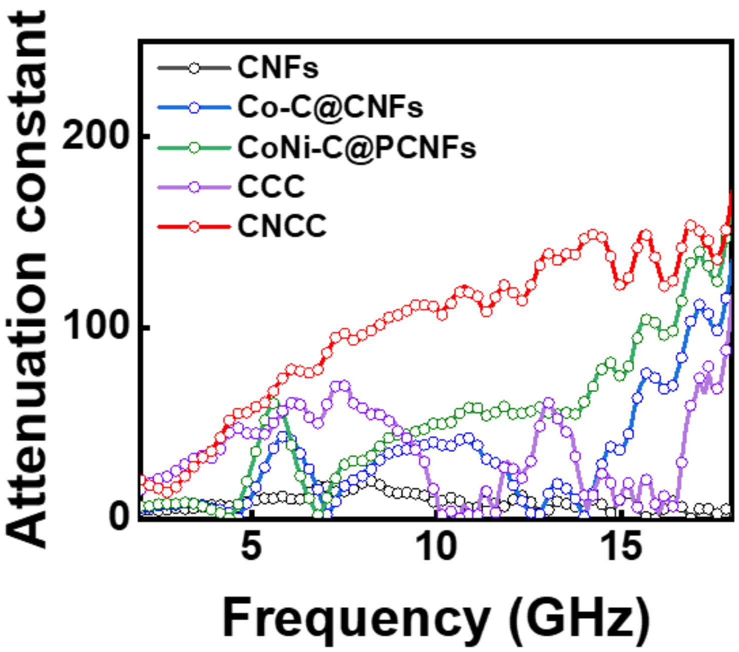

Fig. S14 The attenuation constant curves of all samples

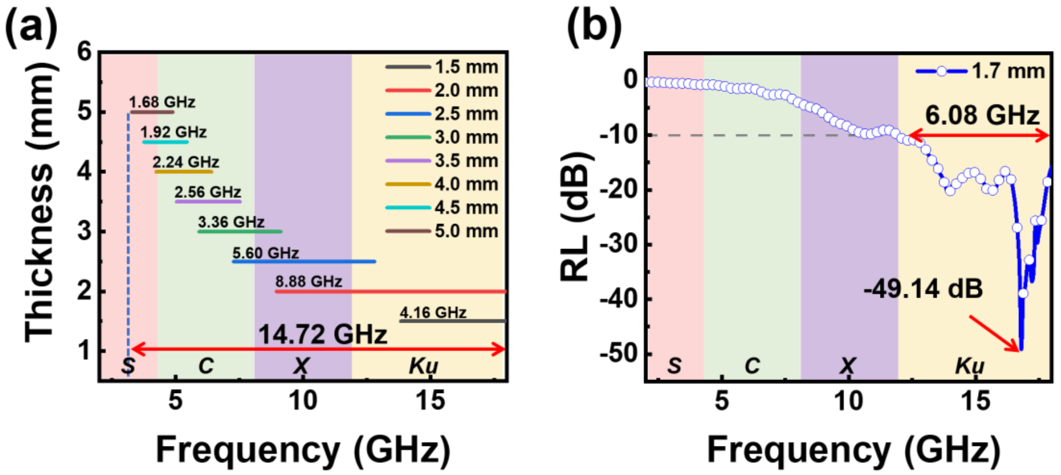

Fig. S15 (**a**) Effective absorption bandwidth values of the sample CNCC at different thicknesses and (**b**) dependence of the reflection loss value on frequency at 1.7 mm

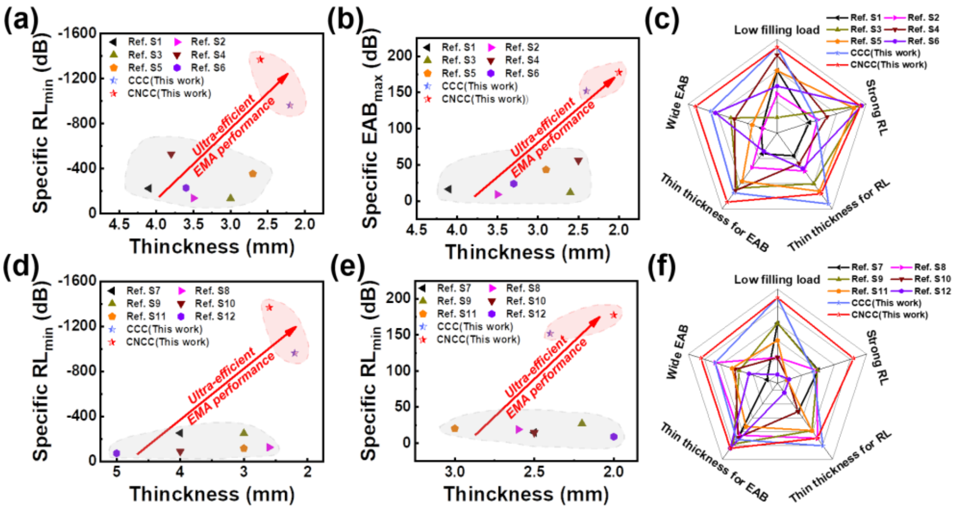

Fig. S16 Ultra-efficient electromagnetic wave absorbers: scatter plots of (**a**) specific RL values versus thickness, (**b**) specific EAB values versus thickness, and (**c**) radar comparison chart of typical 1D composite absorbers reported in the literature and in this work, scatter plots of (**d**) specific RL values versus thickness, (**e**) specific EAB values versus thickness, and (**f**) radar comparison chart of typical MOF-derived composite absorbers reported in the literature and in this work

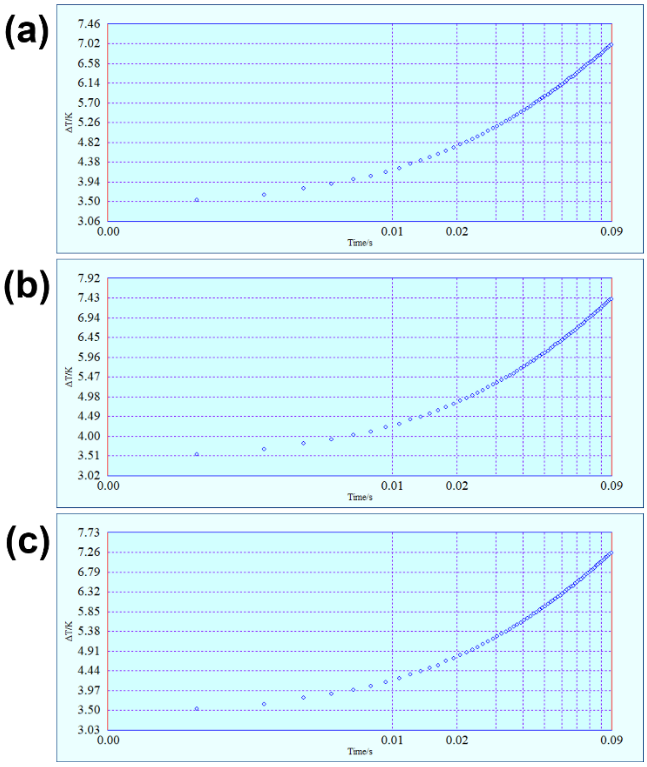

Fig. S17 The temperature difference curves between the upper and lower surfaces of various fibrous membranes on the surface of the thermal conductivity test bench within 0-0.09 s: (**a**) commercial CFs membrane, (**b**) CCC fibrous membrane, and (**c**) CNCC fibrous membrane

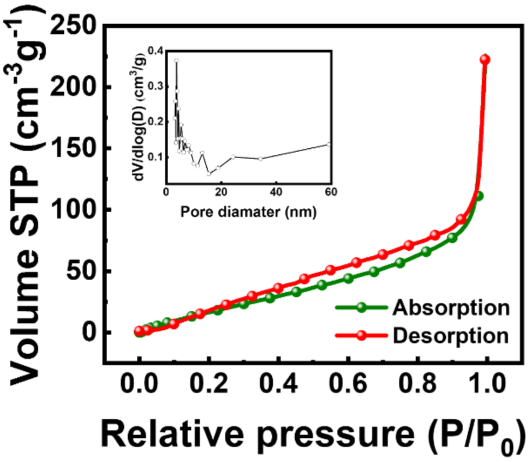

Fig. S18 N_2 adsorption–desorption isotherm curve of CNCC fibrous composite (inset: the corresponding pore size distribution curve)

Table S1 Surface resistance test data for copper tape and various fibrous composites

Sample	Resistance ($10^{-4}\Omega$)					Length (cm)	Width (mm)	Thickness (mm)	Cross-sectional area (mm^2)
Copper strip	17.9	17.6	18.1	17.3	18.2	2	5	0.05	0.25
CNFs	377.1	379.2	378.4	377.6	378.7	2	5	0.10	0.50
CCC	286.2	287.3	286.7	288.1	287.8	2	5	0.11	0.55
CNCC	239.4	237.8	238.7	237.1	238.3	2	5	0.11	0.55

Table S2 Surface element content of various samples (at%)

Sample	C	O	N	Co	Ni	Se
Co-C@CNFs	80.27	7.92	8.06	3.75	0	0
CoNi-C@CNFs	77.56	8.47	6.85	3.51	3.61	0
CCC	81.66	5.28	1.85	5.29	0	5.92
CNCC	72.68	6.08	1.28	4.85	4.99	10.12

Table S3 Comparison of the EMA performance with other typical 1D materials

Sample	Content (wt.%)	RL_{min} (dB)	EAB (GHz)	Refs.
P-CNF/Fe	20	-44.86 (4.1 mm)	3.28 (4.1 mm)	S1 [S1]
HCSS/ZrO_2/SiC	35	-48.6 (3.5 mm)	3.2 (3.5 mm)	S2 [S2]
ZnO/Co nanotubes	50	-68.4 (3.0 mm)	5.9 (2.6 mm)	S3 [S3]
CNT@Ti_3SiC_2@SiC_f	10	-53 (3.8 mm)	5.6 (2.5 mm)	S4 [S4]
Cu_9S_5/C NFs	20	-65.4 (2.7 mm)	4.1 (2.9 mm)	S5 [S5]
MnS/C NFs	30	-68.9 (3.6 mm)	7.2 (3.3 mm)	S6 [S6]
CCC	5	-48.07 (2.2 mm)	7.6 (2.4 mm)	**This work**
CNCC	5	-68.40 (2.6 mm)	8.88 (2.0 mm)	**This work**

Table S4 Comparison of the EMA performance with other typical powdery MOF-derived materials

Sample	Content (wt.%)	RL_{min} (dB)	EAB (GHz)	Refs.
SiC/Ni/NiO/C	20	-50.52 (4.0 mm)	2.96 (2.5 mm)	S7 [S7]
Co/Ni/C	40	-49.8 (2.6 mm)	7.6 (2.6 mm)	S8 [S8]
Ag@C	20	-50.14 (3.0 mm)	5.44 (2.5 mm)	S9 [S9]
Co/C	40	-35.3 (4.0 mm)	5.8 (2.5 mm)	S10 [S10]
NDC/MoS_2	30	-34.94 (3.0 mm)	6.08 (3.0 mm)	S11 [S11]
ZnCo@C@1T-2H-MoS_2	50	-35.83 (5.0 mm)	4.56 (2.0 mm)	S12 [S12]
CCC	5	-48.07 (2.2 mm)	7.6 (2.4 mm)	**This work**
CNCC	5	-68.40 (2.6 mm)	8.88 (2.0 mm)	**This work**

Table S5 Mechanical properties test data of various fibrous membranes

Sample	Width (mm)	Thickness (mm)	Stress (MPa)	Strain (%)
CNFs	20	0.10	4.35	27.8
CCC	20	0.11	5.68	59.3
CNCC	20	0.11	6.16	55.7

Supplementary References

[S1] X.D. Zuo, P. Xu, C.Y. Zhang, M.Z. Li, X.Y. Jiang et al., Porous magnetic carbon nanofibers (P-CNF/Fe) for low-frequency electromagnetic wave absorption synthesized by electrospinning. Ceram. Int. **45**(4), 4474-4481 (2019). https://doi.org/10.1016/j.ceramint.2018.11.127

[S2] B.J. Zhang, Y. Liu, X.L. Li, D. Su, H.M. Ji, Closed-cell ZrO_2/SiC-based composite nanofibers with efficient electromagnetic wave absorption and thermal insulation properties. J. Alloys Compd. **927**, 167036 (2022). https://doi.org/10.1016/j.jallcom.2022.167036

[S3] J. Qiao, D.M. Xu, L.F. Lv, X. Zhang, F.L. Wang et al., Self-assembled ZnO/Co hybrid nanotubes prepared by electrospinning for lightweight and high-performance electromagnetic wave absorption. ACS Appl. Nano Mater. **1**(9), 5297-5306 (2018). https://doi.org/10.1021/acsanm.8b01303

[S4] Y.J. Zou, X.Z. Huang, B.H. Fan, Z.P. Tang, J.H. Zhou et al., Constructing hierarchical Ti_3SiC_2 layer and carbon nanotubes on SiC fibers for enhanced electromagnetic wave absorption. Ceram. Int. **49**(5), 8048-8057 (2023). https://doi.org/10.1016/j.ceramint.2022.10.323

[S5] S.M. Wu, J. Qiao, Y.X. Tang, X. Zhang, X.W. Meng et al., Heterogeneous Cu_9S_5/C nanocomposite fibers with adjustable electromagnetic parameters for efficient electromagnetic absorption. J Colloid Interface Sci. **630**, 47-56 (2023). https://doi.org/10.1016/j.jcis.2022.10.075

[S6] J. Qiao, X. Zhang, C. Liu, Z.H. Zeng, Y.F. Yang et al., Facile synthesis of MnS nanoparticle embedded porous carbon nanocomposite fibers for broadband electromagnetic wave absorption. Carbon **191**, 525-534 (2022). https://doi.org/10.1016/j.carbon.2022.02.024

[S7] R. Yang, J.Q. Yuan, C.H. Yu, K. Yan, Y. Fu et al., Efficient electromagnetic wave absorption by Sic/Ni/Nio/C nanocomposites. J. Alloys Compd. **816**, 152519 (2020). https://doi.org/10.1016/j.jallcom.2019.152519

[S8] G. Liu, J.Q. Tu, C. Wu, Y.J. Fu, C.H. Chu et al., High-yield two-dimensional metal-organic framework derivatives for wideband electromagnetic wave absorption. ACS Appl. Mater. Interfaces **13**(17), 20459-20466 (2021). https://doi.org/10.1021/acsami.1c00281

[S9] Y.H. Song, X.H. Liu, Z.G. Gao, Z.D. Wang, Y.H. Hu et al., Core-shell Ag@C spheres derived from Ag-MOFs with tunable ligand exchanging phase inversion for electromagnetic wave absorption. J Colloid Interface Sci. **620**, 263-272 (2022). https://doi.org/10.1016/j.jcis.2022.04.012

[S10] Y.Y. Lu, Y.T. Wang, H.L. Li, Y. Lin, Z.Y. Jiang et al., MOF-derived porous Co/C nanocomposites with excellent electromagnetic wave absorption properties. ACS Appl. Mater. Interfaces **7**(24), 13604-13611 (2015). https://doi.org/10.1021/acsami.5b03177

[S11] J.H. Luo, M.N. Feng, Z.Y. Dai, C.Y. Jiang, W. Yao et al., MoS_2 wrapped mof-derived n-doped carbon nanocomposite with wideband electromagnetic wave absorption. Nano Res. **15**(7), 5781-5789 (2022). https://doi.org/10.1007/s12274-022-4411-6

[S12] R.R. Yu, Y.H. Xia, X.Y. Pei, D. Liu, S.K. Liu et al., Micro-flower like core-shell structured ZnCo@C@1T-2H-MoS_2 composites for broadband electromagnetic wave absorption and photothermal performance. J Colloid Interface Sci. **622**, 261-271 (2022). https://doi.org/10.1016/j.jcis.2022.01.179
